# Bile salt hydrolase in non-enterotoxigenic *Bacteroides* potentiates colorectal cancer

**DOI:** 10.1038/s41467-023-36089-9

**Published:** 2023-02-10

**Authors:** Lulu Sun, Yi Zhang, Jie Cai, Bipin Rimal, Edson R. Rocha, James P. Coleman, Chenran Zhang, Robert G. Nichols, Yuhong Luo, Bora Kim, Yaozong Chen, Kristopher W. Krausz, Curtis C. Harris, Andrew D. Patterson, Zhipeng Zhang, Shogo Takahashi, Frank J. Gonzalez

**Affiliations:** 1 Laboratory of Metabolism, Center for Cancer Research, National Cancer Institute, Bethesda, MD 20892 USA; 2 Department of General Surgery, Cancer Center, Peking University Third Hospital, Beijing, 100191 China; 3 Center for Molecular Toxicology and Carcinogenesis, Department of Veterinary & Biomedical Sciences, Pennsylvania State University, University Park, PA 16802 USA; 4 Department of Microbiology and Immunology, Brody School of Medicine, East Carolina University, Greenville, NC 27834 USA; 5 Laboratory of Human Carcinogenesis, Center for Cancer Research, National Cancer Institute, Bethesda, MD 20892 USA

**Keywords:** Colon cancer, Colon cancer, Tumour immunology

## Abstract

Bile salt hydrolase (BSH) in *Bacteroides* is considered a potential drug target for obesity-related metabolic diseases, but its involvement in colon tumorigenesis has not been explored. BSH-expressing *Bacteroides* is found at high abundance in the stools of colorectal cancer (CRC) patients  with overweight and in the feces of a high-fat diet (HFD)-induced CRC mouse model. Colonization of *B. fragilis* 638R, a strain with low BSH activity, overexpressing a recombinant *bsh* gene from *B. fragilis* NCTC9343 strain, results in increased unconjugated bile acids in the colon and accelerated progression of CRC under HFD treatment. In the presence of high BSH activity, the resultant elevation of unconjugated deoxycholic acid and lithocholic acid activates the G-protein-coupled bile acid receptor, resulting in increased β-catenin-regulated chemokine (C-C motif) ligand 28 (CCL28) expression in colon tumors. Activation of the β-catenin/CCL28 axis leads to elevated intra-tumoral immunosuppressive CD25^+^FOXP3^+^ T_reg_ cells. Blockade of the β-catenin/CCL28 axis releases the immunosuppression to enhance the intra-tumoral anti-tumor response, which decreases CRC progression under HFD treatment. Pharmacological inhibition of BSH reduces HFD-accelerated CRC progression, coincident with suppression of the β-catenin/CCL28 pathway. These findings provide insights into the pro-carcinogenetic role of *Bacteroides* in obesity-related CRC progression and characterize BSH as a potential target for CRC prevention and treatment.

## Abstract (web summary)

Non-enterotoxigenic *Bacteroides fragilis* (NTBF) is abundant in colorectal cancer (CRC) patients and in a high-fat diet (HFD)-induced CRC model. Here the authors show that bile salt hydrolase-expressing NTBF is enriched in CRC patients with overweight and promotes tumor growth in an HFD-induced CRC mouse model.

## Introduction

CRC, the second most common cause of cancer death in the United States according to 2020 American Cancer Society statistics, is a heterogeneous disease caused by genetic, dietary, and environmental factors^1^. Adenomatous polyposis coli (*APC*) mutation is the initiating event in CRC that is found in over 80% of CRC patients^2^. APC is a negative regulator of the WNT/β-catenin pathway and APC inactivation results in hyperactivation of WNT signaling critical to CRC pathogenesis^3^. CRC mouse models such as *Apc*^min/+^, *Apc*^580S/+^, *Apc*^Δ242/+^, *Cdx2Apc*^f/w^ that mimic human colon carcinogenesis were developed based on the *APC* mutation^4^.

Clinical metagenomics and metabolomics studies have revealed that the gut microbiota is closely correlated with CRC initiation and progression. Polyketide synthetase positive (*pks*^+^) *Escherichia coli*, *Fusobacterium nucleatum* and enterotoxigenic *B. fragilis* (ETBF) have been considered as major microbial drivers of CRC^5^. ETBF is detected in colon mucosa of polyps in patients with familial adenomatous polyposis (FAP)^6^. ETBF secretes an oncogenic toxin called *B. fragilis* fragilysin that increases barrier permeability^7^, augments β-catenin signaling^8^, and induces reactive oxygen species and DNA damage^9^. In multiple murine CRC models, ETBF promotes colon carcinogenesis by increasing the pro-inflammatory response and enhancing epigenetic modifications^10,11^. However, a recent fecal meta-analysis demonstrated no significant difference in *Bft* (encoding *B. fragilis* enterotoxin) abundance between CRC patients and controls^12^. A recent study found that non-enterotoxigenic *B. fragilis* (NTBF), which was found to be *bft* negative, is abundant in polyp mucosa^13^. Thus, it appears that NTBF is also important in the progression of CRC.

BSH catalyzes the gateway reaction leading to the conversion from host-generated conjugated bile acids to microbially-modified unconjugated bile acids and are widely expressed by all major phyla within gut microbiota colonized in human intestine, including Firmicutes, Bacteroidetes, Actinobacteria, and Proteobacteria^14^. In NTBF, BSH was suggested as a potent therapeutic target for metabolic disorders, based on its role in multiple metabolic pathways such as lipid metabolism, cholesterol metabolism, and body weight gain in the host^15–17^. However, its role in CRC progression has not been explored.

In this work, NTBF is found to potentiate CRC progression depending on the high activity of BSH, activation of the β-catenin-CCL28 axis and induction of T_reg_ cells infiltration in colon tumors. These data reveal a pro-carcinogenetic effect of NTBF BSH in colon tumorigenesis and establish BSH as a potential therapeutic target for CRC.

## Results

### BSH-producing *Bacteroides* species were enriched in CRC

**Fig. 1:**
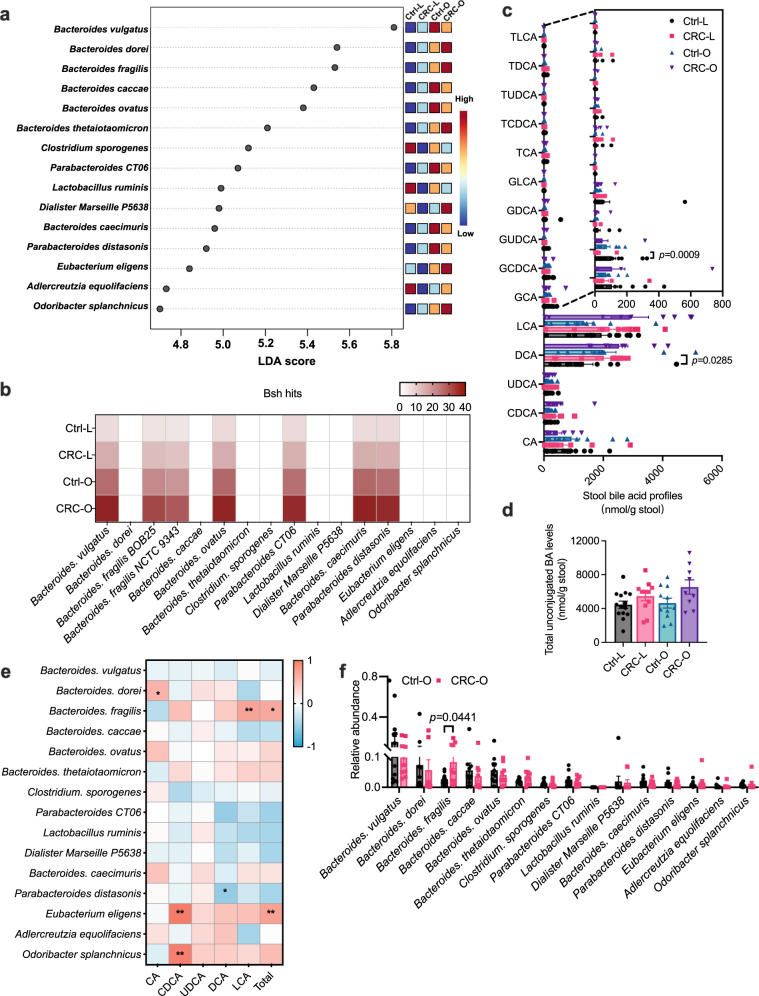
BSH-producing *Bacteroides* species are enriched in CRC patients. **a**–**f** Stool samples were collected from 45 individuals [14 for control lean group (Ctrl-L); 11 for CRC lean group (CRC-L); 11 for control overweight group (Ctrl-O); 9 for CRC overweight group (CRC-O)] for shotgun metagenomics and bile acid analysis. **a** Top 15 species of gut microbiota that led to the differences among four groups. **b** *bsh* hits in the variable gut microbiota species. **c** Stool bile acid profiles. Kruskal–Wallis test with Dunn’s post hoc test. CA: cholic acid; UDCA: ursodeoxycholic acid; T: taurine-; G: glycine-. **d** Total unconjugated bile acids. **e** Heatmap of the correlation between variable gut microbiota species and stool unconjugated bile acid levels in subjects with overweight. Correlation analyses were determined by Spearman’s rank test with two-sided. *p < 0.05, **p < 0.01. The exact *p* values are *p* = 0.0239 (*Bacteroides. dorei* vs CA), *p* = 0.0051 (*Bacteroides. fragilis* vs LCA), *p* = 0.0104 (*Bacteroides. fragilis* vs Total), *p* = 0.0456 (*Parabacteroides distasonis* vs DCA), *p* < 0.0001 (*Eubacterium eligens* vs CDCA), *p* = 0.0043 (*Eubacterium eligens* vs Total), *p* < 0.0001 (*Odoribacter splanchnicus* vs CDCA). **f** Relative abundances of variable gut microbiota in subjects with overweight. Mann–Whitney *U* test with two-sided. Data are presented as mean values +/− SEM in (**c**, **d**, **f**). Source data are provided as a Source Data File for Fig. 1.

High-fat diet (HFD) was found to accelerate tumorigenesis in an obesity-related CRC mouse model with increased bile acid burden^18^. To determine the role of specific functional gut microbiota in obesity-accelerated CRC, metagenomics and bile acid profile analysis were carried out in stool samples collected from healthy controls (Lean/Overweight, Ctrl-L/O) and CRC patients (Lean/Overweight, CRC-L/O) (Supplementary Table 1). No significant differences on ${{{{{\rm{\alpha }}}}}}$-diversity and ${{{{{\rm{\beta }}}}}}$-diversity were found between healthy controls and CRC patients (Supplementary Fig. 1a–d). However, several species of *Bacteroides* contributed to the separation among four groups, with increased abundances in subjects with overweight (Fig. 1a). Among these species, *B. dorei*, *B. fragilis and B. thetaiotaomicron* were further enriched in CRC subjects with overweight (Fig. 1a). Since *Bacteroides* species are a source of BSH that accounts for bile acid deconjugation^19^, *bsh* gene abundances were analyzed in the variable gut microbiota species and bile acid profiles in the stool samples were further quantified. In the variable gut microbiota species, *bsh* genes were mainly detected in several species of *Bacteroides*, and more enriched in CRC patients (Fig. 1b). Chenodeoxycholic acid (CDCA) was increased while the conjugate glycochenodeoxycholic acid (GCDCA) was decreased in CRC patients, indicating that bile acid deconjugation was enhanced by CRC (Fig. 1c). Deoxycholic acid (DCA), lithocholic acid (LCA) and total unconjugated bile acids tended to increase in CRC patients (Fig. 1c, d). Moreover, *B. fragilis* was positively correlated with levels of LCA and total unconjugated bile acids in subjects with overweight, especially in CRC patients with overweight (Fig. 1e and Supplementary Fig. 1e–g). Consistently, the abundance of *B. fragilis* was significantly higher in CRC patients with overweight than that in control subjects with overweight (Fig. 1f and Supplementary Fig. 1h). Although *Eubacterium eligens* and *Odoribacter splanchnicus* were positively related with stool CDCA levels, their abundances between the Ctrl-O and CRC-O groups were similar (Fig. 1e, f).

**Fig. 2:**
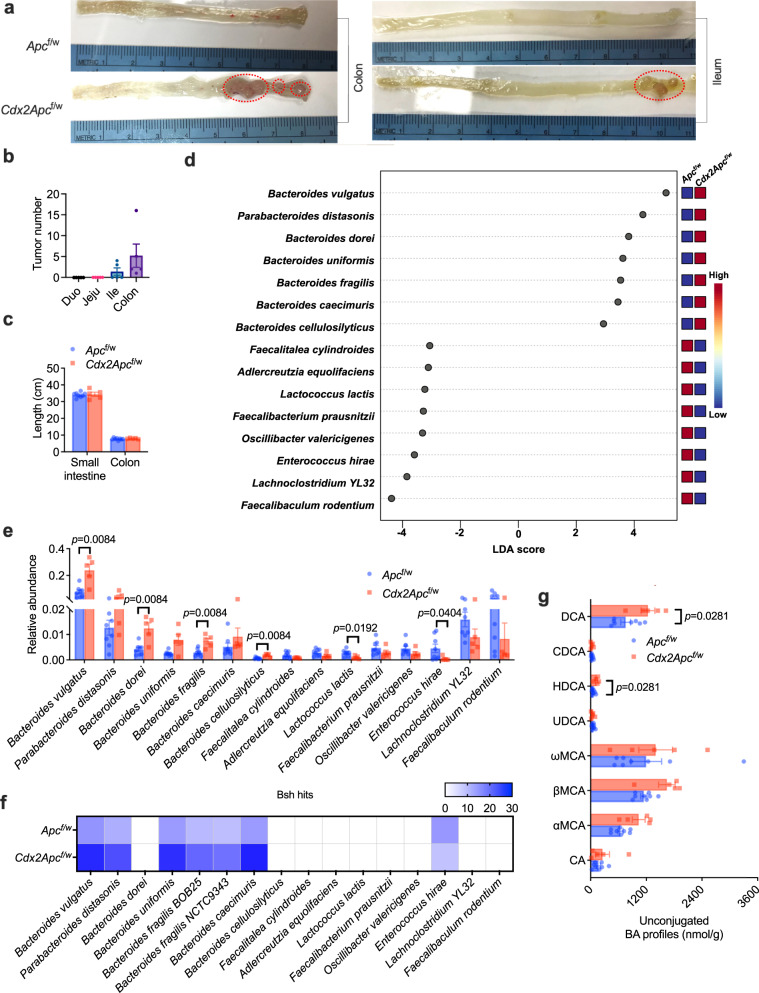
BSH-producing *Bacteroides* species are enriched in a CRC mouse model. **a**–**g** *Apc*^f/w^ and *Cdx*2*Apc*^f/w^ mice were fed a HFD for 12 weeks (*n* = 8 mice for *Apc*^f/w^; *n* = 5 mice for *Cdx*2*Apc*^f/w^). **a** Representative pictures of ileum and colon, revealing the tumor occurrence (in red circle). **b** Tumor numbers in different segments of intestine from 5 *Cdx*2*Apc*^f/w^ mice, Duo: duodenum; Jeju: jejunum; Ile: Ileum. **c** Length of small intestine and colon. **d** Top 15 species of gut microbiota that led to the differences between two groups. **e** Relative abundance of variable gut microbiota species levels. Kruskal–Wallis test with Dunn’s post hoc test. **f** *bsh* hits in the variable gut microbiota species. **g** Unconjugated bile acid profiles in the colon contents. Mann–Whitney *U* test with two-sided. ωMCA: ω-muricholic acid. Data are presented as mean values +/– SEM in (**b**, **c**, **e**, **g**). Source data are provided as a Source Data File for Fig. 2.

*Apc*^min/+^ mice are commonly used in CRC-related studies, but this strain has a major limitation because it does not accurately reflect human colon tumorigenesis, as most tumors develop in the small intestine of Apcmin/+ mice while the vast majority of tumors are found in the colon and rectum in CRC patients (Supplementary Fig. 2a–c). Hence, colon specific *Apc* knockout (*Cdx*2*Apc*^f/w^) mice were used in the current study. To further determine the role of specific gut microbiota and bile acids in obesity-accelerated CRC progression, HFD-fed *Apc*^f/w^ and *Cdx*2*Apc*^f/w^ mouse model was generated. In this model, large tumors were mainly detected in the colon and rectum, with smaller tumors found in the distal ileum (Fig. 2a, b). The tumor occurrence had no significant effect on intestine length, an indicator of colitis (Fig. 2c). Metagenomics was then performed to examine the gut microbiota compositions in these mice. Consistent with clinical data, several species of *Bacteroides* accounted for the differences between the two groups (Fig. 2d and Supplementary Fig. 2d–g). Among *Bacteroides*, the species *B. vulgatus*, *B. dorei*, *B. fragilis* and *B. cellulosilyticus* were present at significantly higher levels in the HFD-fed *Cdx*2*Apc*^f/w^ mice compared to the HFD-fed *Apc*^f/w^ mice (Fig. 2e). In the variable species of the CRC mouse model, *bsh* hits were mainly detected in several species of *Bacteroides*, which were more enriched in the HFD-fed *Cdx2Apc*^f/w^ mice (Fig. 2f). As a result, more abundant unconjugated bile acids were observed in HFD-fed *Cdx*2*Apc*^f/w^ mice, including α/βMCA (α/β-muricholic acid), HDCA (hyodeoxycholic acid) and DCA (Fig. 2g and Supplementary Fig. 2h). Although *Lactococcus lactis* and *Enterococcus hirae* showed lower abundances in HFD-fed *Cdx*2*Apc*^f/w^ mice (Fig. 2e), that was not observed differences in CRC patients (Fig. 1f). These results suggest that CRC is associated with altered homeostasis of the gut microbiota that in turn alters intestinal bile acid composition in CRC.

### Colonization with BSH-producing *Bacteroides* species potentiates CRC progression

**Fig. 3:**
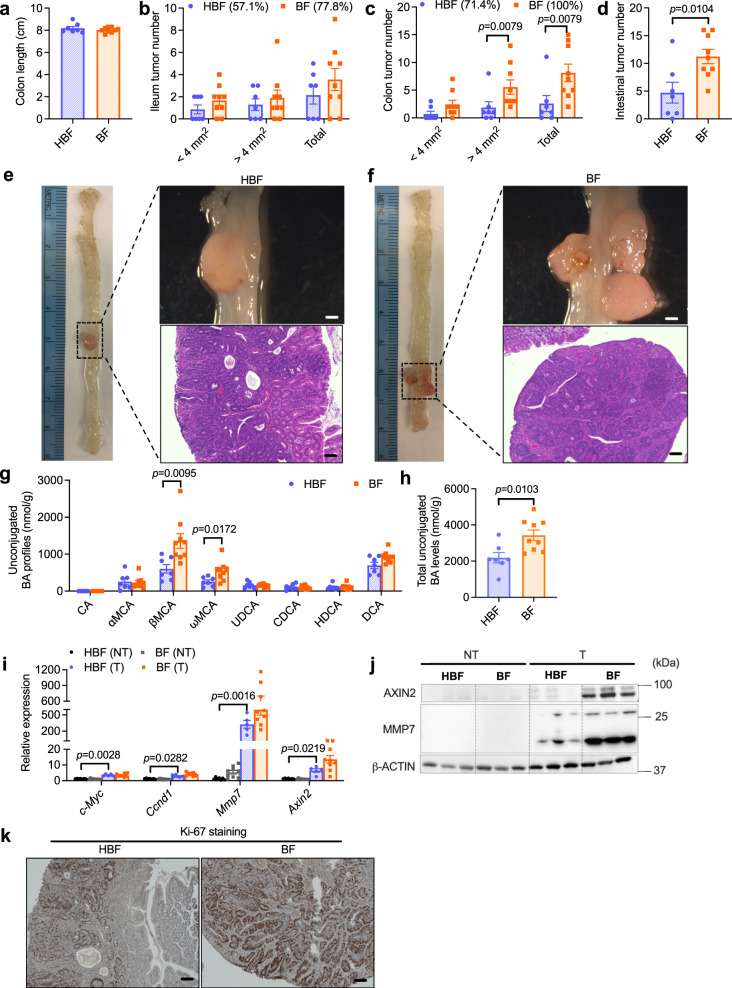
*B. fragilis* NCTC9343 colonization increases CRC progression. **a**–**h** HFD-fed *Cdx*2*Apc*^f/w^ mice were colonized with heat-killed *B. fragilis* 9343 (HBF) or *B. fragilis* 9343 (BF) for 12 weeks (*n* = 7 mice for HBF; *n* = 9 mice for BF). **a** Length of colon. The ileum (**b**) and colon (**c**) tumor incidence, and tumor numbers with different sizes (<4 mm^2^, >4 mm^2^ and the sum of both). Mann–Whitney *U* test with two-sided. **d** Total tumor number in the intestine. Two-tailed Student’s *t* test. **e**, **f** Representative pictures of colon (left), gross images of tumor (top right) in the colon and H&E staining (bottom right) of colon tumor sections. Scale bars: 1.5 mm (top right) and 100 μm (bottom right). **g** Unconjugated bile acid profiles in the colon contents. Mann–Whitney *U* test with two sided. **h** Total unconjugated bile acid levels in the colon contents. Two-tailed Student’s *t* test. **i** Relative mRNA levels of WNT target genes in non-tumor (NT) and tumor (T) colon tissues (*n* = 7 for HBF-NT; *n* = 9 for BF-NT; *n* = 5 for HBF-T; *n* = 9 for BF-T;). Kruskal–Wallis test with Dunn’s post hoc test. **j** WB data of proteins encoded by WNT target genes, *n* = 3 independent samples/group. **k** Representative IHC staining of Ki-67, a proliferation marker (*n* = 5 independent slides for HBF; *n* = 7 independent slides for BF). Scale bars: 100 μm. Data are presented as mean values +/− SEM in (**a**–**d**, **g**–**i**). Source data are provided as a Source Data file for Fig. 3.

To explore the tumorigenic potential of non-enterotoxigenic BSH-producing *Bacteroides species*, HFD-fed *Cdx*2*Apc*^f/w^ mice were colonized with *B. fragilis* NCTC 9343 (BF, NTBF with high BSH activity) or *B. vulgatus* (BV)^16,17^; heat-killed *B. fragilis* 9343 (HBF) or *B. vulgatus* (HBV) were used as controls. To check the colonization efficiency, another commensal bacteria (*Bacteroides xylanisolvens*) was added as a control. *B. fragilis* NCTC 9343 or *B. vulgatus* strains successfully colonized the mouse intestine (Supplementary Fig. 2i). Tumor incidences were increased by BF colonization with 77.8% ileum tumor incidence and 100% colon tumor incidence in the BF group compared with 57.1% ileum tumor incidence and 71.4% colon tumor incidence in the HBF group (Fig. 3a–d). Moreover, BF colonization mainly increased tumor burden in the colon as indicated by increased colon tumor number but no significant changes in ileum tumor numbers (Fig. 3b–d). Gross pictures and histology analysis also revealed that *B. fragilis*-colonized *Cdx*2*Apc*^f/w^ mice developed more advanced CRC (Fig. 3e, f). As a result of increased NTBF BSH, colon bile acid profiles and total colon bile acid load were increased under BF9343 colonization (Fig. 3g, h).

Since bile acids were found to activate WNT signaling^18,20^, the major aberrant pathway in human CRC^2^, the relative expression of several WNT target gene mRNAs were measured, including *Ccnd1* (encoding cyclin D1), *c-Myc* (encoding the MYC proto-oncogene), *Mmp7* (encoding matrix metallopeptidase 7) and *Axin2* (encoding axis inhibition protein 2), in colon non-tumor and tumor tissues of HBF and BF mice. Levels of WNT target gene mRNAs were markedly increased in colon tumor tissues compared with colon non-tumor tissues, especially *Mmp7* and *Axin2* mRNAs (Fig. 3i). BF colonization further increased the relative expression of *Mmp7* and *Axin2* mRNAs (Fig. 3i), and the resultant increased protein levels were further confirmed by western blot analysis (Fig. 3j). Consistently, more advanced proliferation was observed in tumor tissues of BF-colonized *Cdx*2*Apc*^f/w^ mice (Fig. 3k). Similar with *B. fragilis* NCTC 9343, BSH-expressing *B. vulgatus* colonization also potentiated CRC progression with increased tumor incidence and more severe colon tumor burden (Supplementary Fig. 3a–f). Colon unconjugated bile acid profiles and total unconjugated bile acid levels were much higher in the BV group than in the HBV group (Supplementary Fig. 3g, h). Compared with HBV-treated mice, BV-treated mice showed more activated WNT/β-catenin signaling in colon tumor tissues, accompanied by increased cancer cell proliferation (Supplementary Fig. 3i–k). In summary, BSH-expressing *Bacteroides* colonization led to higher colon unconjugated bile acid load, aberrant activation of WNT/β-catenin signaling, and increased CRC progression.

### BSH overexpression in *B. fragilis* increased the colon unconjugated bile acid load and provoked CRC progression

**Fig. 4:**
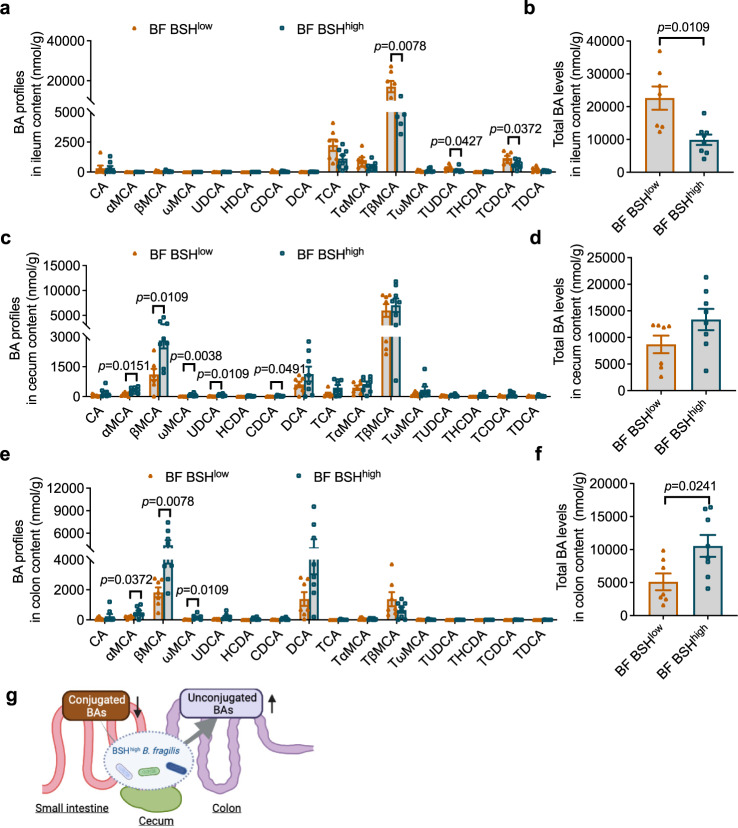
Microbial BSH overexpression in *B. fragilis* facilitates bile acid escape to the colon. **a**–**f** HFD-fed *Cdx*2*Apc*^f/w^ mice were colonized with *B. fragilis* 638R with low BSH activity (BF BSH^low^) or *B. fragilis* 638R with high BSH activity (BF BSH^high^) for 2 weeks (*n* = 7 mice for BF BSH^low^; *n* = 8 mice for BF BSH^high^). Bile acid profiles (**a**) and total bile acid levels (**b**) in the ileum contents. Mann–Whitney *U* test with two-sided (**a**); two-tailed Student’s *t* test (**b**). Bile acid profiles (**c**) and total bile acid levels (**d**) in the cecum contents. Mann–Whitney *U* test with two-sided (**c**). Bile acid profiles (**e**) and total bile acid levels (**f**) in the colon contents. Mann–Whitney *U* test with two-sided (**e**); two-tailed Student’s *t* test (**f**). **g** Schematic diagram showing that conjugated bile acids are deconjugated by BSH-expressing bacteria and escape into colon in the form of unconjugated bile acids. Created with BioRender.com. Data are presented as mean values +/− SEM in a-f. Source data are provided as a Source Data File for Fig. 4.

To further investigate the role of microbial BSH in CRC, the *B. fragilis* 9343 *bsh* gene (BF9343_1433) was inserted into *B. fragilis* 638R (BF BSH^low^), a strain with low endogenous BSH activity against most taurine-conjugated bile acids^21^. *B. fragilis* 638R with inserted BF9343_1433 was defined as BF BSH^high^. To verify BSH overexpression, conjugated bile acids were added to the culture medium of BF BSH^low^ and BF BSH^high^ strains, and the bacteria were incubated overnight. Compared with blank, BF BSH^low^ showed low BSH activity against taurolithocholic acid (TLCA) but had no activity toward other conjugated bile acids (Supplementary Fig. 4a). The BSH and the penicillin V acylase (PVA) are related enzymes and classified as choloylglycine hydrolases (CGH) that belong to the Ntn (N-terminal nucleophile) linear amide C-N hydrolase enzyme superfamily^22^, and the low BSH activity toward TLCA in BF BSH^low^ may be due to the presence of the PVA^21,22^, a possibility that needs to be further confirmed in future studies. With overexpressed BSH, BF BSH^high^ exhibited broad and robust activity toward all the conjugated bile acids in the culture medium (Supplementary Fig. 4a). HFD-fed *Cdx*2*Apc*^f/w^ mice were colonized for two weeks with BF BSH^low^ and BF BSH^high^ (Supplementary Fig. 4b), and ileum, cecum and colon contents were collected for quantification of bile acids in different segments of the intestine. The ileum bile acid pool mainly consisted of conjugated bile acids, with very low levels of unconjugated bile acids (Fig. 4a). BF BSH^high^ colonization resulted in significant decreases of ileum conjugated bile acids and the total bile acid pool (Fig. 4a, b). In cecum contents, the bile acid pool contained both unconjugated and conjugated bile acids, and the unconjugated bile acids were increased by BSH overexpression (Fig. 4c). Total bile acid levels in the cecum tended to increase, but not significantly (Fig. 4d). However, unconjugated bile acids, that predominately made up the colon bile acid pool, were markedly increased by colonization with BF BSH^high^ (Fig. 4e, f). Based on bile acid analysis in different segments of the intestine, BSH-expressing bacteria transformed conjugated bile acids to unconjugated bile acids in the ileum or cecum, which assisted in increased transit of unconjugated bile acids into the colon (Fig. 4g). This is consistent with a previous finding that remodeling of bile acid spatial distribution resulted from microbial BSH overexpression in the lower ileum, cecum, and proximal colon, the main locations for *B. fragilis* colonization^23^.

**Fig. 5:**
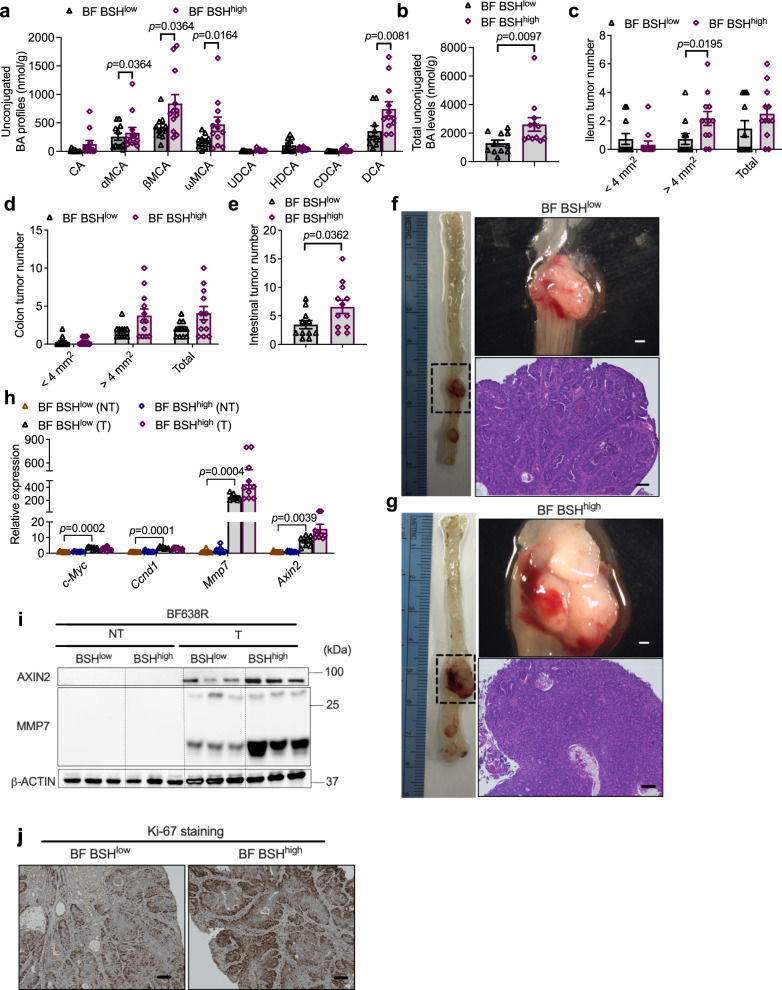
Microbial BSH overexpression in *B. fragilis* potentiates CRC progression. **a**–**h** HFD-fed *Cdx*2*Apc*^f/w^ mice were colonized with BF BSH^low^ or BF BSH^high^ for 12 weeks (*n* = 11 mice for BF BSH^low^; *n* = 12 mice for BF BSH^high^). Unconjugated bile acid profiles (**a**) and total unconjugated bile acid levels (**b**) in the colon contents. Mann–Whitney *U* test with two-sided (**a**). Two-tailed Student’s *t* test (**b**). The ileum (**c**) and colon (**d**) tumor incidence, and tumor numbers with different sizes (<4 mm^2^, >4 mm^2^ and the sum of both. Mann–Whitney *U* test with two-sided. **e** Total tumor numbers in the intestine. Two-tailed Student’s *t* test. **f**, **g** Representative pictures of colon (left), gross images of tumors (top right) in the colon and H&E staining (bottom right) of colon tumor sections. Scale bars: 1.5 mm (top right) and 100 μm (bottom right). **h** Relative mRNA levels of WNT target genes in colon non-tumor (NT) and tumor (T) tissues (*n* = 10 for BF BSH^low^-NT; *n* = 12 for BF BSH^high^-NT; *n* = 8 for BF BSH^low^-T; *n* = 10 for BF BSH^high^-T). Kruskal–Wallis test with Dunn’s post hoc test. **i** WB data of proteins involved in WNT signaling (*n* = 3 independent samples/group). **j** Representative IHC staining of Ki-67 (*n* = 8 independent slides for BF BSH^low^; *n* = 9 independent slides for BF BSH^high^). Scale bars: 100 μm. Data are presented as mean values +/− SEM in (**a**–**e**, **h**). Source data are provided as a Source Data File for Fig. 5.

A long-term colonization of BF BSH^low^ and BF BSH^high^ in HFD-fed *Cdx*2*Apc*^f/w^ mice was then conducted to determine whether BSH overexpression influences CRC progression. Consistently, long-term BF BSH^high^ colonization significantly increased unconjugated bile acid profiles in the colon (Fig. 5a, b). HFD-fed *Cdx*2*Apc*^f/w^ mice with BF BSH^high^ colonization exhibited more severe colon tumor burden, without a significant change in colon length (Fig. 5c–g and Supplementary Fig. 4c). Consistent with the cancer potentiation phenotype, BSH overexpression led to more profound activation of WNT/${{{{{\rm{\beta }}}}}}$-catenin signaling, as well as cancer cell proliferation in colon tumor tissues (Fig. 5h–j). Taken together, these findings indicate that BSH overexpression in NTBF promotes the conversion of conjugated bile acids to unconjugated bile acids, which further escape into the colon (Fig. 4g) and accelerate CRC progression, accompanied by further activation of WNT/${{{{{\rm{\beta }}}}}}$-catenin signaling.

### Microbial BSH overexpression induced intra-tumoral immunosuppressive properties

**Fig. 6:**
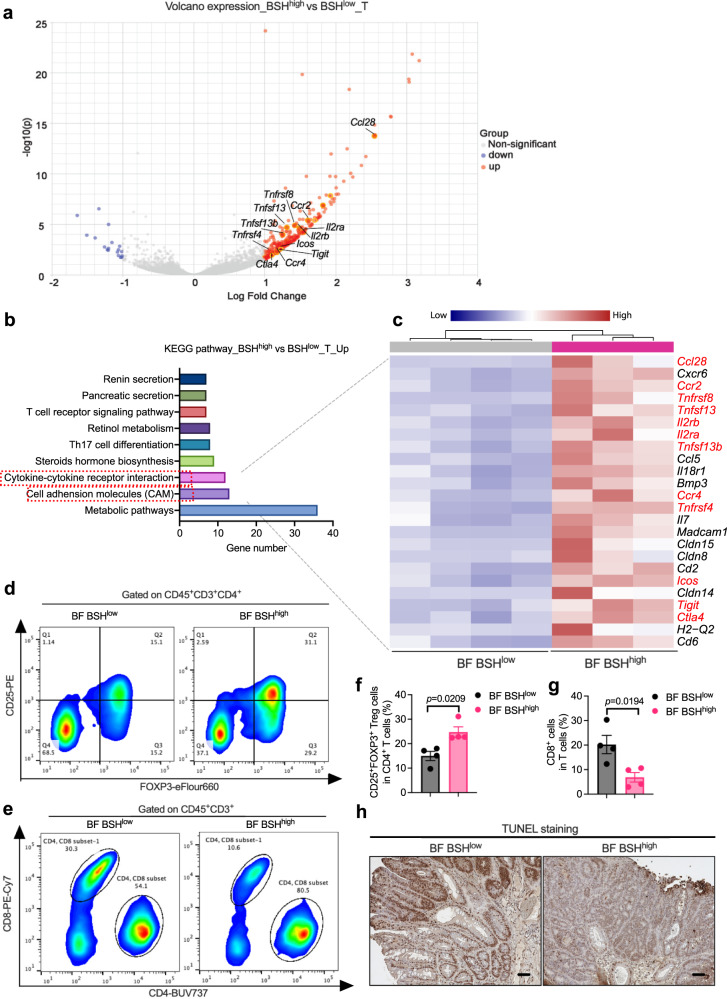
Microbial BSH overexpression in *B. fragilis* induced intra-tumoral immunosuppressive properties. **a**–**c** HFD-fed *Cdx*2*Apc*^f/w^ mice were colonized with BF BSH^low^ or BF BSH^high^ for 12 weeks, from which the colon non-tumor (NT) and tumor (T) tissues were applied for mRNA sequencing (pooled sample sizes: *n* = 4 independent samples for BF BSH^low^_NT; *n* = 4 independent samples for BF BSH^high^_NT; *n* = 4 independent samples for BF BSH^low^_T; *n* = 3 independent samples for BF BSH^high^_T). **a** Volcano plot showing the upregulated and downregulated genes in colon tumor tissues. Red dots (upregulated) and blue dots (downregulated) were identified as significantly changed mRNAs with multiple comparisons adjusted *P* value < 0.05 and |logFC| > 1. Two-tailed Student’s *t* test. **b** Significantly (multiple comparisons adjusted *P* value < 0.05) enriched pathway terms of upregulated genes in colon tumor tissues under microbial BSH overexpression (BSH^high^ vs BSH^low^) indicated by KEGG pathway analysis. Two-tailed Student’s t test. **c** Heatmap showing all the upregulated genes that involved in cytokine–cytokine receptor interaction and cell adhesion molecules (CAM) under microbial BSH overexpression (BSH^high^ vs BSH^low^). The genes in red were identified as markers of T_reg_ cells as reported. **d**–**g** HFD-fed *Cdx*2*Apc*^f/w^ mice were colonized with BF BSH^low^ or BF BSH^high^ for 12 weeks, from which the colon tumor tissues were applied for flow cytometry analysis (*n* = 4 independent samples/group). Flow cytometry analysis determined the FOXP3^+^CD25^+^ T_reg_ cells (**d**) and CD8^+^ T cells (**e**) in colon tumor tissues. The portion of FOXP3^+^CD25^+^ T_re**g**_ cells in CD4^+^ T cells (**f**) and CD8^+^ T cells in total T cells (**g**). Two-tailed Student’s *t* test. **h** HFD-fed *Cdx*2*Apc*^f/w^ mice were colonized with BF BSH^low^ or BF BSH^high^ for 12 weeks (*n* = 11 mice for BF BSH^low^; *n* = 12 mice for BF BSH^high^). Representative IHC staining of TUNEL, an apoptosis marker (*n* = 8 independent slides for BF BSH^low^; *n* = 9 independent slides for BF BSH^high^). Scale bars: 100 μm. Data are presented as mean values +/− SEM in (**f**, **g**). Source data are provided as a Source Data File for Fig. 6.

To comprehensively determine the underlying alterations resulting from BSH overexpression in CRC, colon non-tumor and tumor tissues were collected from HFD-fed *Cdx*2*Apc*^f/w^ mice after 12-week BF (BSH^low^ or BSH^high^) colonization, and RNA-seq was performed (Supplementary Data 3). The PCA score plot of the RNA-seq data revealed a close clustering gene expression profile in colon non-tumor tissues of the two groups (BSH^low^ and BSH^high^) (Supplementary Fig. 5a). Moreover, a volcano plot showed that there was only one significantly upregulated gene in BSH-overexpressed mice compared with control mice (Supplementary Fig. 5c). These data indicate that BSH overexpression has little influence on gene expression in colon non-tumor tissues. However, an obvious separation was observed in colon tumor tissues from BF BSH^low^ and BF BSH^high^-treated *Cdx*2*Apc*^f/w^ mice (Supplementary Fig. 5b). The volcano plot included 303 mRNAs (287 upregulated and 16 downregulated) that were differentially expressed in colon tumor tissues between the two groups (Fig. 6a). Most of the significantly different gene mRNAs were increased by BSH overexpression, indicating that some intra-tumoral tumorigenic pathways were activated by BF BSH. To verify this hypothesis, the upregulated pathways during CRC progression were screened by KEGG pathway analysis (Fig. 6b). The significantly upregulated genes in the colon tumor tissues of BF BSH^high^ colonized mice found in the heatmaps were involved in cytokine–cytokine receptor interaction and CAM pathways (Fig. 6b, c). Most of these genes were regulatory T (T_reg_) cell surface markers, such as *Icos* (encoding inducible costimulatory), *Tigit* (encoding T cell immunoglobulin with ITIM domain), *Ctla4* (encoding cytotoxic T-lymphocyte-associated protein 4), *Il2ra* (also called *Cd25*, encoding interleukin 2 receptor subunit α), *Il2rb* (encoding interleukin 2 receptor subunit β), and *Ccr2*/*4* (encoding C-C motif chemokine receptor 2/4), or related to the activation of T_reg_ cells, such as the tumor necrosis factor receptor superfamily (*Tnfrsf*)^24–26^. To quantify the T_reg_ cell populations, single cell suspensions of the colon tumors were prepared after a 12-week BF BSH^low^ or BF BSH^high^ colonization, and flow cytometry analysis was conducted (Supplementary Fig. 6a). Consistent with the mRNA analysis, there were a higher portion of colon CD25^+^FOXP3^+^ T_reg_ cells in CD4^+^ T cells of BF BSH^high^-colonized mice than that in BF BSH^low^-colonized mice (Fig. 6d, f). As a result, intra-tumoral anti-tumor immune response was suppressed by BF BSH as indicated by a decreased cytotoxic CD8^+^ T cells population and TUNEL positive apoptotic cancer cells (Fig. 6e, g, h and Supplementary Fig. 6b).

### CCL28-induced immunosuppressive T_reg_ cell accumulation accounted for BF BSH-accelerated CRC advancement

**Fig. 7:**
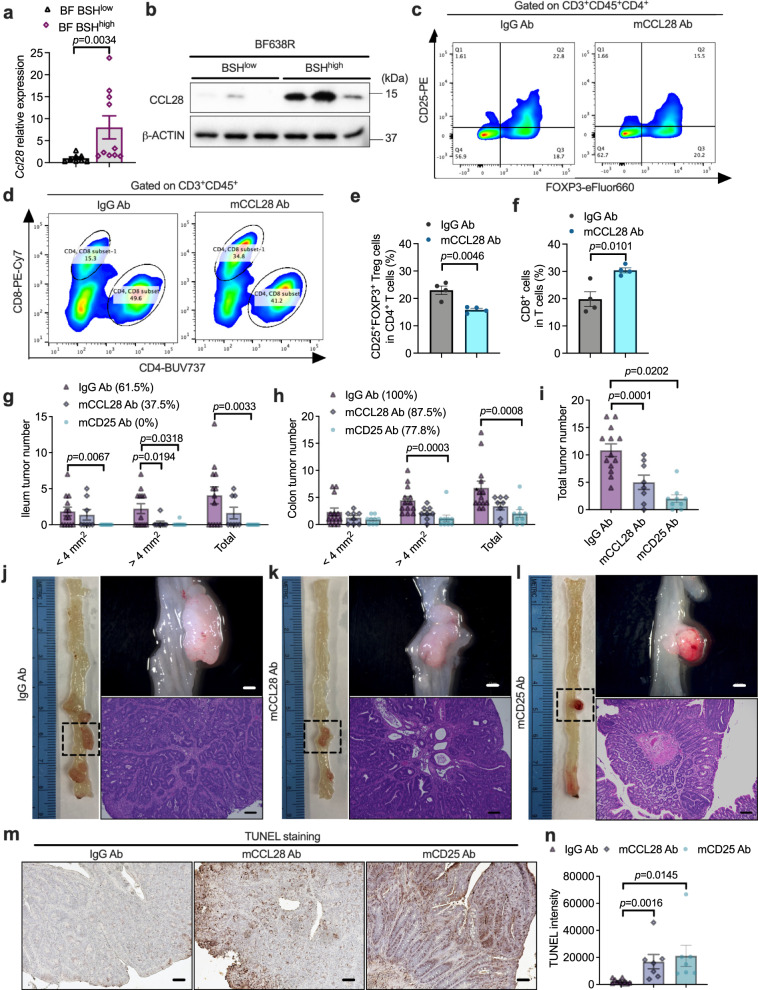
CCL28-induced accumulation of T_reg_ cells contributed to *B. fragilis*-accelerated CRC progression. **a** HFD-fed *Cdx*2*Apc*^f/w^ mice that were colonized with BF BSH^low^ or BF BSH^high^ for 12 weeks (*n* = 8 mice for BF BSH^low^; *n* = 10 mice for BF BSH^high^). *Ccl28* relative expression in colon tumor tissues. Two-tailed Student’s *t* test. **b** CCL28 protein levels (*n* = 3 independent samples/group). **c**–**f** *Cdx*2*Apc*^f/w^ mice were fed with HFD for 10 weeks, and then the mice were colonized with BF BSH^high^ and injected (i.p.) with IgG or mCCL28 neutralizing antibody for another 2 weeks. The colon tumor tissues were used for flow cytometry analysis (*n* = 4 independent samples/group). Flow cytometry analysis determined the FOXP^+^CD25^+^ T_reg_ cells (**c**) and CD8^+^ T cells (**d**) in colon tumor tissues. The portion of FOXP^+^CD25^+^ T_reg_ cells in CD4^+^ T cells (**e**) and CD8^+^ T cells in total T cells (**f**). Two-tailed Student’s *t* test. **g**–**n** HFD-fed *Cdx*2*Apc*^f/w^ mice were fed with HFD for 6 weeks, and then colonized with BF BSH^high^ and injected (i.p.) with IgG, mCCL28 Ab or mCD25 Ab neutralizing antibody for another 6 weeks (*n* = 13 mice for IgG Ab; *n* = 8 mice for mCCL28 Ab; *n* = 9 mice for mCD25 Ab). The ileum (**g**) and colon (**h**) tumor incidence, and tumor numbers with different sizes (<4 mm^2^, >4 mm^2^ and the sum of both). Mann–Whitney *U* test with two-sided. **i** Total tumor number **i**n the intestine. Two-tailed Student’s *t* test. **j**–**l** Representa*t*ive pictures of colon (left), gross images of tumors (top right) in the colon and H&E staining (bottom right) of colon tumor sections. Scale bars: 1.5 mm (top right) and 100 μm (bottom right). Representative IHC staining (**m**) and quantification (**n**) of TUNEL (*n* = 10 in dependent slides for IgG Ab; *n* = 7 independent slides for mCCL28 Ab; *n* = 7 independent slides for mCD25 Ab). Scale bars: 100 μm. Two-tailed Student’s *t* test. Data are presen*t*ed as mean values +/− SEM in (**a**, **e**–**i**, **n**). Source data are provided as a Source Data File for Fig. 7.

Of note, *Ccl28*, encoding CCL28, a major chemokine involved in T_reg_ cell trafficking, showed a marked induction in colon tumor tissues from BF BSH^high^-colonized mice (Figs. 6a, c, 7a, b). To explore the role of CCL28-induced immunosuppression in BF BSH-accelerated CRC, anti-CCL28 or anti-CD25 neutralizing antibody were applied to block CCL28 or CD25^+^FOXP3^+^ T_reg_ cells^27,28^. Either CCL28 or CD25 blockade decreased T_reg_ cell accumulation, resulting in the increase of cytotoxic CD8^+^ T cells in colon tumor tissues (Fig. 7c–f and Supplementary Fig. 6c–f). The antibody treatments did not disturb bile acid metabolism (Supplementary Fig. 6g–j). Importantly, the BF BSH-accelerated CRC progression was ameliorated by anti-CCL28 or anti-CD25 antibody treatment, which mainly resulted from enhanced cancer cell apoptosis (Fig. 7g–n and Supplementary Fig. 6k).

**Fig. 8:**
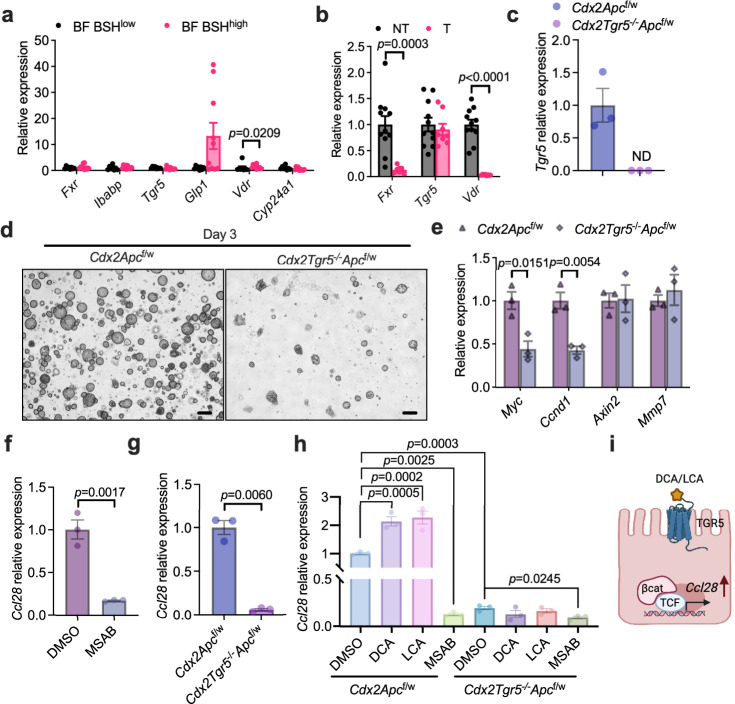
DCA/LCA activates TGR5 signaling that regulates *Ccl28* expression in the presence of β-catenin. **a** HFD-fed *Cdx*2*Apc*^f/w^ mice were colonized with BF BSH^low^ or BF BSH^high^ for 12 weeks (*n* = 8 mice for BF BSH^low^; *n* = 10 mice for BF BSH^high^). Relative expression of *Fxr*, *Tgr5* and *Vdr*, as well as their target genes, in colon tumor tissues. Mann–Whitney *U* test with two-sided. **b** Relative expression of *Fxr*, *Tgr5* and *Vdr* in colon non-tumor (NT) and tumor (T) tissues (*n* = 11 independent samples for NT; *n* = 8 independent samples for T). Mann–Whitney *U* test with two-sided. **c**–**e** *Cdx*2*Apc*^f/w^ and *Cdx*2*Tgr5*^−/−^*Apc*^f/w^ mice were fed with HFD for over 10 weeks, and then the colon tumors were used for colon organoid isolation and further culturing (*n* = 3 independent samples /group). **c** Relative expression of *Tgr5*. **d** Representative images of colon tumor-derived organoids at day 3 after passage. Scale bars: 200 μm. **e** Relative expression of ${{{{{\rm{\beta }}}}}}$-catenin target genes. Mann–Whitney *U* test with two-sided. **f** Isolated colon tumor organoids were treated with DMSO or MSAB (10 μM, a *β*-catenin protein degrader) overnight (*n* = 3 technical replicates/group). Relative expression of *Ccl28*. Two-tailed Student’s *t* test. **g**, **h** *Cdx*2*Apc*^f/w^ and *Cdx*2*Tgr5*^−/−^*Apc*^f/w^ mice were fed with a HFD for over 10 weeks, and then the colon tumors were used for colon organoid isolation and further culturing (*n* = 3 tech*n*ical replicates/group). **g** Relative expression of *Ccl28*. Two-tailed Student’s *t* test. **h** Relative expression of *Ccl28* under DMSO, DCA (100 μM), LCA (100 μM), MSAB treatment overnight. Kruskal–Wallis test with Dunn’s post hoc test. **i** Diagram that uncovers that DCA/LCA-TGR5 signaling regulates *Ccl28* expression in the presence of ${{{{{\rm{\beta }}}}}}$-catenin in colon cancer cell. Created with BioRender.com. Data are presented as mean values +/− SEM in (**a**–**c**, **e**–**h**). Source data are provided as a Source Data File for Fig. 8.

Bile acids activate WNT/${{{{{\rm{\beta }}}}}}$-catenin signaling, and *Ccl28* was identified as a β-catenin target gene in gastric cancer cells^18,20,29^. Due to the expression of murine *Cyp2c70*, most of the CDCA is metabolized to hydrophilic muricholic acids (MCAs) in mice instead of the production of LCA in humans^30,31^. Although some muricholic acids were increased due to the BSH-expressing *Bacteroides* in the CRC mouse model, it was not clinically relevant. By combining human and mouse data, a potential DCA/LCA downstream mechanism was explored. As reported, DCA and LCA could activate farnesoid X receptor (FXR) and G protein-coupled bile acid receptor 1 (GPBAR1, also called TGR5), and LCA could activate vitamin D receptor (VDR)^32^. In HFD-fed *Cdx2Apc*^f/w^ mice, BSH^high^ BF activated the TGR5 pathway in colon tumor tissues but did not increase FXR and VDR signaling (Fig. 8a). Compared with non-tumor tissues, the relative levels of *Fxr* and *Vdr* mRNAs were decreased in tumor tissues, which may result in their low transcriptional activation activities (Fig. 8b). To determine the role of TGR5 in the β-catenin/CCL28 axis, colon tumor organoids were isolated from HFD-fed *Cdx2Apc*^f/w^ and *Cdx2Tgr5*^−/−^*Apc*^f/w^ mice (Fig. 8c). Loss of TGR5 resulted in fewer and smaller colon organoids derived from single colon cancer cells (Fig. 8d). Consistently, lower expression of β-catenin target genes was observed in TGR5-deficient colon tumor organoids, including *Myc* and *Ccnd1* (Fig. 8e). Either degradation of β-catenin or absence of TGR5 decreased the relative expression of *Ccl28* in colon tumor organoids (Fig. 8f–h). Hence, the induction of *Ccl28* under microbial BSH overexpression mainly resulted from the activation of WNT/${{{{{\rm{\beta }}}}}}$-catenin signaling (Fig. 8i). In summary, BSH in NTBF induced the intra-tumoral immunosuppressive properties. Moreover, CCL28 plays a vital role in trafficking the CD25^+^FOXP3^+^ T_reg_ cells under BSH overexpression.

### Pharmacological inhibition of microbial BSH relieved CRC progression

**Fig. 9:**
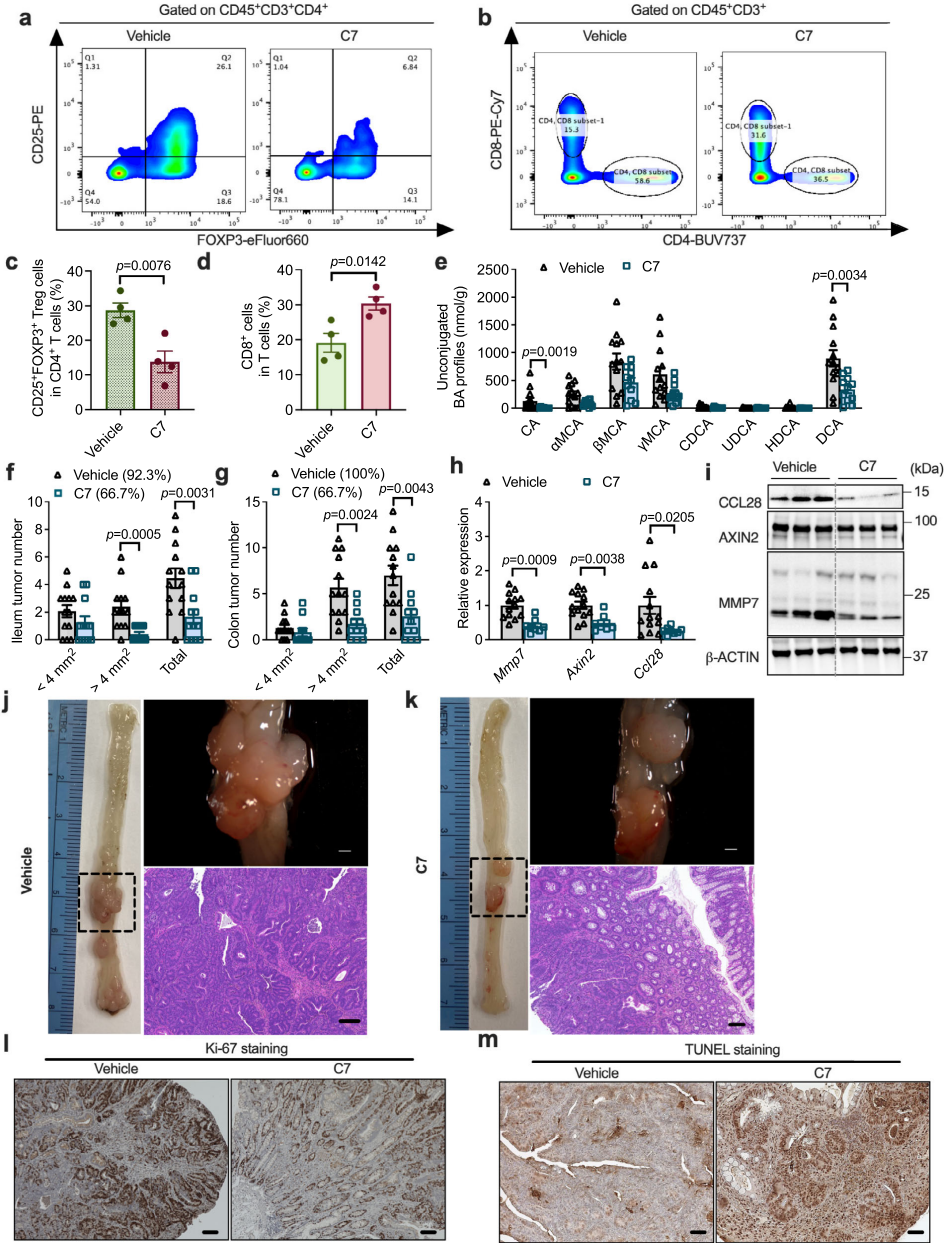
Pharmacological inhibition of microbial BSH alleviated colon cancer. **a**–**d** *Cdx*2*Apc*^f/w^ mice were fed with HFD for 10 weeks, and then the mice were colonized with BF BSH^high^ and fed with HFD or HFD with compound 7 for another 2 weeks. The colon tumor tissues were used for flow cytometry analysis (*n* = 4 independent samples/group). Flow cytometry analysis determined the FOXP^+^CD25^+^ T_reg_ cells (**a**) and CD8^+^ T cells (**b**) in colon tumor tissues. The portion of FOXP^+^CD25^+^ T_reg_ cells in CD4^+^ T cells (**c**) and CD8^+^ T cells in total T cells (**d**). Two-tailed Student’s *t* test. **e**–**k** Under BF BSH^high^ colonization, *Cdx*2*Apc*^f/w^ mice were fed with HFD or HFD with compound 7 for 12 weeks (*n* = 13 mice for Vehicle; *n* = 11 mice for C7). **e** Unconjugated bile acid profiles in colon contents. Mann–Whitney *U* test with two-sided. The ileum (**f**) and colon (**g**) tumor incidence, and tumor numbers with different sizes (<4 mm^2^, >4 mm^2^ and the sum of both). Mann–Whitney *U* test with two-sided. **h** Relative mRNA levels of WNT target genes and *Ccl28* in colon tumor tissues. Mann–Whitney *U* test with two-sided. **i** WB data of CCL28 and proteins involved in WNT signaling (*n* = 3 independent samples/group). **j**, **k** Representative pictures of colon (left), gross images of tumor (top right) in the colon and H&E staining (bottom right) of colon tumor sections. Scale bars: 1.5 mm (top right) and 100 μm (bottom right). Representative IHC staining of Ki-67 (**l**) and TUNEL (**m**). *n* = 9 independent slides for Vehicle; *n* = 9 independent slides for C7. Scale bars: 100 μm. Data are presented as mean values +/− SEM in (**c**–**h**). Source data are provided as a Source Data File for Fig. 9.

To test the potential of microbial BSH as a drug target for CRC treatment, a BSH inhibitor, Compound 7 (C7) was used^33^. The BSH inhibitory effects of C7 were first verified in a culture medium of BF9343 (Supplementary Fig. 7a). To explore the in vivo effects of C7, *Cdx*2*Apc*^f/w^ mice were fed a HFD for ten weeks and then divided into two groups that were colonized with BF BSH^high^ and fed a standard HFD or HFD-containing C7 for another two weeks. The accumulation of CD25^+^FOXP3^+^ T_reg_ cells was decreased and cytotoxic CD8^+^ T cells was increased by administration of C7 in the colon tissues of BF BSH^high^-colonized mice (Fig. 9a–d). Then, in *Cdx*2*Apc*^f/w^ mice colonized with BF BSH^high^, long-term C7-containing HFD feeding was performed, which showed no liver toxicity (Supplementary Fig. 7b, c). With BSH overexpression, colon unconjugated bile acid overload was mitigated by C7 treatment (Fig. 9e, Supplementary Fig. 7d). With C7 supplementation, less tumor burden and decreased WNT/*β*-catenin signaling were observed in the intestine, with no change in colon length (Fig. 9f–i and Supplementary Fig. 7e, f). Histological analysis also revealed that the CRC progression was delayed by C7 treatment in HFD-fed *Cdx*2*Apc*^f/w^ mice colonized with BF BSH^high^, as indicated by decreased proliferation and increased apoptosis of cancer cells (Fig. 9j–m and Supplementary Fig. 7g). In vitro, C7 treatment had no direct effect on the β-catenin/CCL28 axis in cultured colon tumor-derived organoids (Supplementary Fig. 7h). These data suggest the potential of C7 for CRC treatment mainly targets microbial BSH.

## Discussion

In the present study, enrichment of BSH-expressing *Bacteroides* was found to exert pro-tumorigenic effects throughout CRC progression in two CRC mouse models. With colonization of a genetically modified *B. fragilis* strain with high BSH, it was discovered that BSH overexpression accelerated CRC progression by allowing excess bile acids to escape into the colon. Bile acids promoted WNT signaling and upregulated CCL28 expression in a β-catenin-dependent manner in colon tumor tissues. Under BSH overexpression, more CD25^+^FOXP3^+^ T_reg_ cells penetrated the colon due to greater CCL28 trafficking. Pharmacological suppression of microbial BSH with C7 was shown to be effective in slowing the progression of CRC.

Multi-omics approaches, such as LC-MS metabolomics and metagenomic sequencing, have revealed a strong relationship between gut microbiota, microbial metabolites, and CRC progression in the clinic^12,34–39^. CRC animal models and correlation analysis of human CRC data revealed possible colorectal carcinogenic effects of some bacteria, including *Fusobacterium nucleatum*, *Pks*^+^ *Escherichia coli*, and *Bft*^+^ *B. fragilis*^10,40–42^. *B. fragilis* was consistently enriched in HFD-fed *Cdx*2*Apc*^f/w^ mice in the present study. Other *Bacteroides* species, in addition to *B. fragilis*, also have higher abundances in HFD-fed *Cdx*2*Apc*^f/w^ mice than in HFD-fed *Apc*^f/w^ mice. Increased unconjugated bile acid levels in the colon contents of HFD-fed *Cdx*2*Apc*^f/w^ mice support the view that microbial BSH activity is enhanced during CRC progression. *Bacteroides* BSH was shown to play a role in metabolic diseases like diabetes and polycystic ovary syndrome that are caused by particular conjugated bile acids in the small intestine^16,17^. However, it is unclear how *Bacteroides* BSH alters colon bile acid metabolism throughout the progression of CRC. Bile acids are produced in the liver and released into the duodenum in the form of bile^43^. The portal vein reabsorbs and recycles the majority of bile acids in the small intestine, with a minor fraction escaping into the colon^43^. In the current study, microbial BSH overexpression was found to facilitate the transit of bile acids from the small intestine to the colon. An earlier tracking investigation^23^ revealed that remodeling of the bile acid spatial distribution was correlated with the colonization site of *B. fragilis* at the ileum-colon junction. DCA and LCA are not a bile acid produced by microbial BSH genes and *Bacteroides* don’t have other genes involved in DCA/LCA production, but an increase of DCA was observed after colonizing BSH^high^ BF and higher LCA in CRC patients with overweight. Hence, the increase of BSH^high^ BF might initiate bacterial bile acid metabolism, and the tumor-promoting effects need the synergistic action of secondary bile acid-producing bacteria. Several bile acid metabolites were identified as CRC promotors, such as DCA, LCA and taurine-β-muricholic acid (TβMCA). Increased intestinal DCA, LCA, and TβMCA concentrations trigger cellular responses such as activation of the WNT/β-catenin and NF-κB signaling pathways^18^ that result in DNA oxidative damage and enhanced mitotic activities^44,45^, as well as activation of intrinsic apoptotic pathways such as mitochondrial oxidative stress, cytochrome C release, and cytosolic caspases^45,46^. Consistently, aberrant activation of WNT/β-catenin signaling in colon tumor tissues was observed under microbial BSH overexpression accompanied by increased unconjugated bile acid loads in the colon, especially DCA and βMCA. Many CRC studies exclusively focused on serum bile acid levels, but there are limitations reflected in the in situ changes initiated by altered microbial activities. In the present study, bile acid remodeling in the contents of different intestinal segments was investigated, revealing the change of local bile acid deconjugation in the ileum-colon junction in CRC.

RNA-seq was further conducted to explore the mechanism by which microbial BSH potentiated CRC progression. By colonization of *B. fragilis* with high BSH activity, gene expression profiles in non-tumor tissues revealed no alterations. However, in colon tumor tissues, BSH overexpression increased the relative expression of multiple genes involved in cytokine–cytokine receptor interaction and CAM pathways, consistently upregulated throughout CRC progression. This indicates that microbial BSH has little significant influence on the initiation of CRC but accelerates the progression of CRC through altering the intra-tumoral microenvironment. The majority of the elevated genes in the immunity-related cytokine–cytokine receptor interaction and CAM pathways were identified as surface indicators or activators of FOXP3^+^ T_reg_ cells^24–26^. Abundant T_reg_ cell infiltration into tumors has been considered as a strong predictor for poor clinical outcomes in several cancers, while the role of T_reg_ cells in CRC is controversial^47^. Recently, tumor infiltrating FOXP3^+^ T_reg_ cells were found to have functionally different subpopulations associated with distinct CRC prognosis^48^, indicating that more comprehensive markers should be used to identify different subtypes of intra-tumor FOXP3^+^ Treg cells. Regardless, depleting FOXP3^+^ T_reg_ cells from tumor tissues is an effective treatment for CRC and other malignancies, primarily because it alleviates immunosuppression^49–52^. In the current study, microbial BSH overexpression enhanced immunosuppression, as evidenced by a larger proportion of FOXP3^+^ T_reg_ cells in colon CD4^+^ T cells.

CCL28, an important chemokine for the recruitment of intra-tumoral T_reg_ cells, promotes tumor tolerance and angiogenesis under hypoxia^53^. RNA-seq analysis revealed that microbial BSH overexpression enhanced the relative expression of *Ccl28* in colon tumor tissue, suggesting a role for BSH in regulating the intra-tumoral immune response. The increase of intra-tumor FOXP3^+^ T_reg_ cells caused by BF BSH^high^ colonization was partially reversed by blocking CCL28, confirming a vital role for CCL28 in recruiting intra-tumor FOXP3^+^ T_reg_ cells during CRC progression. The upregulation of *Ccl28* by microbial BSH overexpression resulted from bile acid-induced activation of WNT signaling, since *Ccl28* was positively regulated by β-catenin. CCL28 was identified as a β-catenin target gene in gastric cancer cells, and blocking the β-catenin/CCL28 axis suppresses T_reg_ cell infiltration and gastric cancer progression^29^. In addition to the trafficking effects of CCL28, gut microbiota-derived bile acid metabolites are considered as important regulators of T_reg_ cell differentiation^54,55^. Moreover, the bile acid pool is essential for the maintenance of colon FOXP3^+^ T_reg_ cells, especially RORγ^+^ FOXP3^+^ T_reg_ cells^56^. Rather than 7α-hydroxysteroid dehydrogenase (7α-HSDH), microbial BSH in *Bacteroides* accounts for the induction of RORγ^+^ FOXP3^+^ T_reg_ cells in the colon^56^. Further studies are warranted to explore the existence of pathways other than CCL28 that are involved in the modification of the immune response caused by microbial BSH.

Several BSH inhibitors were developed in order to investigate the possibility of inhibiting microbial BSH as a treatment for metabolic disorders^15,16,33,57^. The potential of microbial BSH as a therapeutic target in CRC progression was tested using C7, a BSH inhibitor that covalently binds the enzyme. In vitro and in vivo, C7 demonstrated a potent reduction of microbial BSH activity, and long-term HFD treatment with C7 alleviated the colon bile acid burden, immunosuppression, and CRC progression. This study determined the link between *Bacteroides* BSH and CRC, as well as the underlying mechanism that accounts for the pro-tumorigenic activity of BSH from *Bacteroides*. *Bacteroides* BSH inhibition may be a promising therapeutic target for the therapy of CRC.

## Methods

### Ethics statement

This human sample collection conformed to the ethical principles outlined by the Declaration of Helsinki and ethical approval for this study was obtained from the Ethics Committee of Peking University Third Hospital (IRB00006761-LM2022557). All subjects have signed informed consent forms prior to enrollment in the study. No participant compensation was offered. All mice were maintained in a specific pathogen-free (SPF) environment, and all animal protocols (protocol numbers: LM-027 and LM-092) for mouse experimentation were approved by the National Cancer Institute (NCI) Animal Care and Use Committees of National Institutes of Health (NIH).

### Human subject

Healthy individuals and individuals who were diagnosed with CRC by colonoscopy and/or undergone colorectal surgery at the Peking University Third Hospital were enrolled. According to the cut-offs of BMI provided by the World Health Organization (WHO), individuals of BMI ≥ 25 kg/m^2^ belong to overweight group, and individuals of BMI < 25 kg/m^2^ belong to the lean group. Exclusion criteria are as follows: (1) Patients used antibiotics, probiotics preoperative four weeks; (2) Patients with a history of inflammatory bowel disease (Crohn’s disease, ulcerative colitis); (3) Patients with a history of other malignant tumors; (4) Patients with a history of intestinal resection and (5) Patients receiving neoadjuvant therapy before surgery. Finally, 45 individuals (14 for control lean group; 11 for control overweight group; 11 for CRC lean group; 9 for CRC overweight group) were included in the study. The information related to health status (i.e., age, sex, BMI and biochemical indicators) were collected (Supplementary Table 1). The blood samples were centrifuged at 825 × *g* for 15 min after 30 min at room temperature. Separate the supernatant to obtain the serum samples and stored at −80 °C. The levels of blood biochemical indicators (i.e., TG, TC, AST, and ALT) were measured using an autoanalyzer (BioTek Instruments 800TS). Feces samples were collected and frozen in dry ice immediately and stored at −80 °C for subsequent metagenomic and metabolomic tests.

### Mice

For this study, 6- to 8-week-old C57BL/6J (WT), C57BL/6J-*Apc*^*Min*^/J (*Apc*^min/+^, The Jackson Laboratory, #002020), C57BL/6-*Apc*^*tm1Tyj*^/J (*Apc*^f/w^, The Jackson Laboratory, #009045) and B6.Cg-Tg (CDX2-cre)101Erf (The Jackson Laboratory, #009350) were applied. The mice were randomly divided into different groups, housed 3–5 per cage and maintained under standard laboratory conditions (the light from 08:00 to 20:00, the temperature at 21–24 °C and the humidity at 40–70%) with free access to a 60% HFD (Bio-Serv, Cat#S3282) and water at the NCI, NIH. To obtain colon-specific APC mutation, *Apc*^f/w^ the mice were crossed with B6.Cg-Tg (CDX2-cre)101Erf mice harboring the Cre recombinase under control of the *Cdx2* promoter. For screening the gut microbiota during CRC progression, 6- to 8-week-old male *Apc*^f/w^ and *Cdx*2*Apc*^f/w^ mice were fed a 60% HFD for 12 weeks. For bacteria colonization, 6- to 8-week-old male *Cdx*2*Apc*^f/w^ mice were given three-day antibiotic cocktail water [1 mg/mL neomycin (Millipore Sigma, #N1876), 1 mg/mL streptomycin (Millipore Sigma, #S19137), and 1 mg/mL bacitracin (Millipore Sigma, #B0125)], and then the mice were treated with 2 × 10^8^ CFUs of bacteria in 200 μL of sterile anaerobic PBS by gavage every three days. For short-term CCL28 blockage, 6- to 8-week-old male *Cdx*2*Apc*^f/w^ mice were pretreated with 60% HFD for 10 weeks, and then were colonized with BF BSH^high^ and received injections of 50 mg/kg IgG (clone#HRPN, Bio X Cell, Cat#BP0088) or mCCL28 Ab (clone#134306, R&D systems, Cat#MAB533) twice a week for another 2 weeks. For long-term CCL28/T_reg_ cell blockage, 6- to 8-week-old male *Cdx*2*Apc*^f/w^ mice were pretreated with 60% HFD for 6 weeks, and then were colonized with BF BSH^high^ and received injections of IgG (25 mg/kg), mCCL28 Ab (50 mg/kg) or mCD25 Ab (25 mg/kg, clone#PC-61.5.3, Bio X Cell, Cat#BP0012) twice a week for another 6 weeks. For short-term C7 treatment, 6- to 8-week-old male *Cdx*2*Apc*^f/w^ mice were pretreated with 60% HFD for 10 weeks, and then colonized with BF BSH^high^ and fed a 60% HFD or 60% HFD containing 1 mg/g C7 (TargetMol), which equates to 10 mg/kg/d consumption. For long-term C7 treatment, 6- to 8-week-old male *Cdx*2*Apc*^f/w^ mice were colonized with BF BSH^high^ and fed a 60% HFD or 60% HFD containing C7 for 12 weeks.

### Bacterial DNA extraction, 16S rRNA sequencing and shotgun metagenomics

For clinical subjects, DNA (700 ng/sample) was extracted from stool samples for shotgun metagenomics^58^. The genomic DNA was randomly sheared into short fragments. The obtained fragments were end repaired, A-tailed and further ligated with Illumina adapter. The fragments with adapters were PCR amplified, size selected, and purified. Library insert size was evaluated by Agilent Bioanalyzer 2100 system and the library was checked with real-time PCR for quantification. According to the manufacturer’s recommendations and index codes, HiSeq 250 PE clustering suite was adopted to cluster the index coded samples by the cBot clustering generation system, while the Illumina Nova 6000 platform was chosen to sequence, and 150 bp paired-end reads were generated.

Mice were put into the metabolic cages for 24 h to collect feces samples. The feces samples were used to measure the gut microbiota composition using shotgun metagenomics. Genomic DNA was extracted from feces using the E.Z.N.A. stool DNA kit (Omega Bio-Tek, #D4015) according to the manufacturer’s instructions. DNA concentrations were measured by NanoDrop. For shotgun metagenomics, the samples were submitted to Pennsylvania State University Genomics Core Facility (University Park, PA) for NextSeq Mid-Output 150 × 150 paired end sequencing. Obtained demultiplexed reads were checked for quality using fastqc^59^. To remove the host reads from the metagenomic sequences, trimmed reads were aligned genome of C57BL/6J mice and filtered using Kneaddata version v0.7.4. Clean metagenomic sequence reads were analyzed using the Kraken 2 version 2.0.8-beta^60^ taxonomic sequence classification approach on the standard Kraken database comprising all complete bacterial, viral, and archeal genomes in RefSeq. The abundance of the various species was estimated using Bracken.

To remove the host reads from the metagenomic sequences, trimmed reads were aligned with the genome of C57BL/6J mice and filtered using Kneaddata^61^. For functional gene analysis, a local Diamond database^62^ was created using all the Kegg genes involved in bile acid metabolism^63^. Filtered reads were aligned to the local database with Diamond version 0.9.36 with a search flag, sensitive. Post alignment, high quality alignments (identity > 50%, and e-value <1e−4, and bit-score >40) for each gene was counted. These counts were then normalized to total number of reads and converted to counts-per-million.

### Colonization efficiency detection

Cecum contents were collected after *Bacteroides* species colonization and genomic DNA extracted using the QIAamp fast DNA stool mini kit (Qiagen, #51604). NanoDrop was used to determine the genomic DNA concentrations. With *Bacteroides* species-specific primers that target 16S rRNA genes, genomic DNA at 10 ng/µL was subjected to quantitative real-time PCR analysis. The relative levels of specific 16S rRNA gene was normalized with the universal 16S rRNA gene for gut microbiota. The primers are listed in Supplementary Table 2.

### Bile acid quantification

Samples (25 mg) were added 500 µL acetonitrile containing internal standard [0.5 µM D4-UDCA (Cayman, #21892)]. The above tissue samples were homogenized, and centrifugated at 18000 g for 10 min and the supernatants (100 µL for tissue, 150 µL for serum) were concentrated to nearly dryness. The concentrated residues were reconstituted using 200 µL of mixture of methanol and water (3:7, v/v) and transferred into injection vial/96 plate for instrument analysis. Bile acid metabolites were identified and quantified by an Acquity® UPLC/G2Si QTOFMS system (Waters Corp.) with an electrospray ionization (ESI) source. An Acquity® BEH C18 column (100 × 2.1 mm; internal diameter, 1.7 mm; Waters Corp.) was used for chromatographic separation. The mobile phase consisted of a mixture of 0.1% formic acid in water (A) and 0.1% formic acid in acetonitrile (B). The gradient elution was started from 80% A for 4 min, decreased linearly to 60% A over 11 min, to 40% A over the next 5 min, to 10% A for the succeeding 1 min, and finally increased to 80% A for 4 min to re-equilibrate the column. Column temperature was maintained at 45 °C, and the flow rate was 0.4 ml/min. Mass spectrometry was operated in the negative mode. A mass range of m/z 50–1200 was acquired. Bile acid quantification was analyzed by TargetLynx in MassLynx Version4.2.

### Microscopic and histological analysis

Ileums and colons were collected and flushed with PBS and the colon lengths measured from the cecum-colon junction to the end of rectum. The ileums and colons were opened longitudinally, and gross pictures were taken before further microscopic analysis. Tumor numbers were counted under a stereo microscope equipped with a Jenoptik camera and television monitor, and the tumor size were further analyzed by a ProgRes CapturePro version 2.10.0.1, and all the tumor sizes didn’t exceed the maximal tumor size (2 cm in any dimension) permitted by NCI Animal Care and Use Committees of NIH. For histological analysis, the colon tumors were fixed with 10% formalin for 24–48 hours, and then the fixed tissues were paraffin embedded and further processed for standard H&E staining.

For IHC staining, tissue sections were deparaffinized for 5 min three times at room temperature in fresh xylene, then washed for 5 min three times at room temperature in 100% ethanol. Slides were immersed in graded ethanol washes (90%, 80%, 70%, and water) for 5 min each at room temperature in order to rehydrate the samples. Using the DeadEndTM Colorimetric TUNEL System (Promega, # G7130), TUNEL assays were carried out in accordance with the kit’s instructions. For TUNEL signal intensity and area measurements, all images were taken by Keyence BZ-X710 under the same settings, and signal intensity was measured on Fiji software (ImageJ version 1.53t; National Institutes of Health) after background subtraction^64^. For Ki-67 staining, Recombinant Anti-Ki67 antibody (clone#SP6, Abcam, Cat#ab16667, 1:100 dilution) and VECTASTAIN® Elite® ABC-HRP Kit, Peroxidase (Rabbit IgG) (Vector, #PK-6101) were used. Briefly, deparaffinized slides were heated in 1 × Antigen Unmasking Solution, Citrate-Based (Vector laboratories, #H-3300-250) until for 10 min for antigen recovery. To quench endogenous peroxidase activity, the slides were incubated in 3% H_2_O_2_ solution for 15 min. After 60 min of incubation with regular goat serum, the sections were incubated for 15 min with the Avidin Solution and subsequently for 15 min with the Biotin Solution (Vector, Avidin/Biotin Blocking Kit, #SP-2001). Slides were then incubated with Ki-67 antibody diluted in PBS buffer with 2.5% normal goat serum at 4 °C overnight. After incubating with diluted biotinylated secondary antibody for 30 min, the slides were then incubated with prepared VECTASTAIN Elite ABC Reagent for 30 min. ImmPACT® DAB Peroxidase Substrate (Vector, #SK-4105) were used for the HRP detection system.

### Quantitative real-time PCR

The tissues were collected and stored in −80 °C before processing. The tissues were lysed with Trizol^TM^ Reagent (Invitrogen, #15596026), and the total RNA were extracted by phenol/chloroform. cDNA was synthesized from 1 μg total RNA using qSript cDNA SuperMix (Quantabio, #95048). The primer sequences applied in this study were listed in the Supplementary Table 2. The relative level of each mRNA was calculated as fold change compared with the control group after normalizing with *Actb* or *Gapdh* mRNAs.

### Western blot analysis

The colon non-tumor and tumor tissues were homogenized and lysed in the RIPA lysis buffer supplemented with protease inhibitor cocktail. The protein concentrations were quantified with the Pierce^TM^ BCA protein assay kit (ThermoFisher, #23225). The protein samples were further separated by SDS-polyacrylamide gel electrophoresis and transferred to polyvinylfluoride membranes. The membranes were incubated with antibodies against rabbit anti-mouse polyclonal anti-AXIN2 (Abcam, Cat#ab32197, 1:1000 dilution), rabbit anti-mouse monoclonal MMP7 (clone#D4H5, Cell signaling, Cat#3801, 1:1000 dilution), rabbit anti-mouse monoclonal anti-ACTB (clone#13E5, Cell signaling, Cat#4670, 1:1000 dilution), and rabbit anti-mouse monoclonal anti-CCL28 (clone#G-2, Santa Cruz, Cat#sc-376654, 1:500 dilution). Bands of specific proteins were visualized under image analyzer after incubation with the SuperSignal™ west dura extended duration substrate (ThermoFisher, #34075).

### Bacteria culture and colonization in mice

*B. fragilis* 9343, *B. fragilis* 638 R or *B. vulgatus* was streaked onto a BHI (Brain-Heart Infusion medium) plate in a 37 °C anaerobic incubator for 2 d. A single colony was selected and further inoculated into 5 ml PPS (supplemented Proteose Peptone media) as seed cultures, which were incubated with shaking overnight at 37 °C. The seed cultures were further expanded and cultivated and split into 15 ml corning tubes flash frozen with liquid nitrogen before storage at −80 °C. The bacteria were recovered by shaking at 37 °C for 30 min, and a bacteria suspension was made with 5 × 10^8^ CFU/ml PBS for oral delivery in mice. After three-day antibiotic cocktail treatment, the mice were given 200 μL bacterial suspension or the same dose of heat-killed (100 °C, 10 min) bacteria suspension by gavage every three days.

### Construction of the BF638R strain carrying the BF9343 bsh gene

A 1569 bp DNA fragment containing 182 bp upstream of the ATG start codon and 200 bp downstream the stop codon of the BF9343_1433 monocistronic operon was amplified from *B. fragilis* ATCC 25285 chromosome (isogenic to NCTC 9343) using primers CGH-XbaI-FOR and CGH-PstI-REV. The 1569 bp DNA amplified fragment was digested with XbaI and PstI enzymes and cloned into the XbaI/PstI sites of pNBU2-*bla*-*ermGb* vector^65^. The new plasmid, BER-300, was mobilized from *E. coli* S17-1 λpir^66^ into *B. fragilis* 638R, Rif^r^^67^ by biparental mating as previously described^68^ to construct BER-182. The presence of the *bsh* gene confers positive conjugated bile acid activity in BER-182 (BF BSH^high^). The BER-154 strain, *B. fragilis* 638R carrying pNBU-*bla*-*ermGb* empty vector, was used as negative conjugated bile acid activity control strain (BF BSH^low^). The detailed materials are listed in Supplementary Table 3.

### Hydrolysis efficiency of conjugated bile acids under cultured Bacteroides

Bacterial seed cultures were inoculated into 2 ml PPS medium, which contained the taurine-conjugated bile acid mixture (5 μM each) of taurocholic acid (TCA, Millipore Sigma, #86339), TβMCA (Cayman, #20289), taurochenodeoxycholic acid (TCDCA, Millipore Sigma, #T6260), THDCA (Millipore Sigma, #T0682), taurodeoxycholic acid (TDCA, Millipore Sigma, #T0875) and taurolithocholic acid (TLCA, Millipore Sigma, #T7515). These cultures were then grown overnight anaerobically at 37 °C. The entire culture was subjected to the bile acid measurement. To verify the inhibitory effects of BSH inhibitor, 10 μM C7 was added to the culture medium during inoculation. Hydrolysis efficacy was determined by the percentage of remaining conjugated bile acids in the bacterial culture medium, which was normalized with blank controls defined as 100%.

### RNA-seq and pathway analysis

Total RNA of colon non-tumor and tumor tissues was extracted by use of the RNeasy plus mini kit (Qiagen, #74134). After extraction, the RNA samples were subjected to 4200 TapeStation analysis for evaluating the purity and concentration. mRNA-Seq samples were pooled and sequenced on NextSeq2000 using Illumina TruSeq Stranded mRNA Library Prep and paired-end sequencing by the National Cancer Institute Core Sequencing Facility. Reads of the samples were trimmed for adapters and low-quality bases using Cutadapt before alignment with the reference genome (mm10) and the annotated transcripts using STAR. The mapping statistics are calculated using Picard software. Library complexity is measured in terms of unique fragments in the mapped reads using Picard’s MarkDuplicate utility. In addition, gene expression quantification analysis was performed for all samples using STAR/RSEM tools. The raw counts were further uploaded to the online NetworkAnalyst platform (https://www.networkanalyst.ca) for gene expression and KEGG pathway analysis.

### Flow cytometry analysis

Colon with tumors was harvested, opened longitudinally, and flushed with cold PBS. The tumors were collected and further washed in cold colon buffer^69^ and cut into small fragments that were transferred into gentleMACS™ C Tubes (Miltenyi Biotec, #130-093-237) with 10 mL pre-warm colon buffer containing 100 U/mL collagenase E (Millipore Sigma, #C2193). Cell dissociation was performed with gentleMACS™ Octo Dissociator (Miltenyi Biotec). Isolated single cells (~1 × 10^6^) were resuspended in FACS buffer and stained with LIVE/DEAD^TM^ fixable yellow dye (ThermoFisher, #L34959) and cell surface markers (1:100), including rabbit anti-mouse monoclonal CD45-FITC (clone#30-F11, eBioscience, Cat#11-0451-82), hamster anti-mouse monoclonal CD3e-BUV395 (clone#145-2C11, BD Biosciences, Cat#563565), rat anti-mouse monoclonal CD4-BUV737 (clone#RM4-5, BD Biosciences, Cat#612843), rat anti-mouse monoclonal CD8a-PE-Cy7 (clone#53-6.7, BD Biosciences, Cat#552877), rat anti-mouse monoclonal CD25-PE (clone#3C7, BD Biosciences, Cat #553075). Before further staining with nuclear FOXP3 (1:50) using rat anti-mouse monoclonal FOXP3 (clone#PCH101, eFluor 660, eBioscience, Cat#50-4776-42) antibody, the cells were fixed and permeabilized with eBioscience^TM^ FOPX3/transcription factor staining buffer set (ThermoFisher, #00-5523-00). Flow cytometry analysis was conducted by LSRFortessa SORP I (BD Biosciences).

### Colon tumor organoids isolation and culture

Colons with tumors were collected, flushed with cold PBS, and opened longitudinally. The colon tumor organoids were isolated and cultured as previously described^70^. In brief, colon tumors were excised with scissors under microscopy. The colon tumor tissues were further cut into small pieces and incubated with EDTA chelation buffer for 60 min on ice to remove the normal intestinal epithelia. After chelation, the colon tumor tissues were further incubated with digestion buffer containing 200 U/ml type IV collagenase (STEMCELL technologies, #07247) and 125 μg/ml type II dispase (Millipore Sigma, #D4693) for 120 min at 37 °C. The single tumor cell suspensions were resuspended with a cold Matrigel (Corning, #354230)-basal culture medium mixture, seeded and polymerized in a 24-well plate. The isolated single tumor cells form colon tumor formed organoids in the basal culture medium containing 50 ng/mL murine EGF (R&D systems, #2028-EG).

### Data analysis

No data were excluded during the data analysis, and Microsoft Excel version 16.68 and GraphPad Prism version 9.0 was used to collect and analyze the statistical data. All the data were shown as the mean ± SEM, with two-tailed Student’s t test, Mann–Whitney U test, one-way ANOVA with Tukey’s post hoc test and Kruskal–Wallis test followed by Dunn’s test for significance determination. *P* value < 0.05 was considered as significant. Flow cytometry data analysis was performed with Flow Jo Version 10 (BD Biosciences).

### Reporting summary

Further information on research design is available in the Nature Portfolio Reporting Summary linked to this article.

## Supplementary information

Supplementary Information

Description of Additional Supplementary Files

Supplementary Data 1

Supplementary Data 2

Supplementary Data 3

Reporting Summary

## Data Availability

Bulk mRNA-seq output is listed in Supplementary Data 3 and the original sequencing data set has been deposited in GEO database under accession code GSE190298. Human and mouse shotgun metagenomics outputs are listed in Supplementary Data 1 and 2, and the sequencing datasets have been uploaded to the public database in National Library of Medicine under accession codes PRJNA786913 for mouse and PRJNA881471 for human. The remaining data are available within the Article, Supplementary Information or Source Data file. Source data are provided with this paper.

## Source data

Source Data

## Code availability

The code used for the analyses performed in this study is accessible at GitHub (https://github.com/bipinrimal/Lulu_BfragBSH/) and archived at Zenodo^71^ (10.5281/zenodo.7460215).
