# Supplementary material for: Bile salt hydrolase in non-enterotoxigenic Bacteroides potentiates colorectal cancer
Source: Nat Commun. 2023 Feb 10;14:755. doi: 10.1038/s41467-023-36089-9 (PMC9918522; doi:10.1038/s41467-023-36089-9)
Supplement: Supplementary file 1 — Supplementary Information [file 41467_2023_36089_MOESM1_ESM.pdf]

Supplementary Information file

Bile salt hydrolase in non-enterotoxigenic *Bacteroides* potentiates colorectal cancer

Lulu Sun[1,6], Yi Zhang[2,6], Jie Cai[1], Bipin Rimal[3], Edson R. Rocha[4], James P. Coleman[4], Chenran Zhang[5], Robert G. Nichols[3], Yuhong Luo[1], Bora Kim[1], Yaozong Chen[1], Kristopher W. Krausz[1], Curtis C. Harris[5], Andrew D. Patterson[3*], Zhipeng Zhang[2*], Shogo Takahashi[1*], Frank J. Gonzalez[1*]

[1]Laboratory of Metabolism, Center for Cancer Research, National Cancer Institute, Bethesda, MD 20892, USA.

[2]Department of General Surgery, Cancer Center, Peking University Third Hospital, Beijing 100191, China.

[3]Center for Molecular Toxicology and Carcinogenesis, Department of Veterinary & Biomedical Sciences, Pennsylvania State University, University Park, PA 16802, USA.

[4]Department of Microbiology and Immunology, Brody School of Medicine, East Carolina University, Greenville, NC 27834, USA.

[5]Laboratory of Human Carcinogenesis, Center for Cancer Research, National Cancer Institute, Bethesda, MD 20892, USA.

[6]These authors contributed equally.

*Correspondence: F.J.G. (gonzalef@mail.nih.gov); S.T. (shogo.takahashi@nih.gov); Z.Z. (zhangzhipeng06@126.com); A.D.P. (adp117@psu.edu)

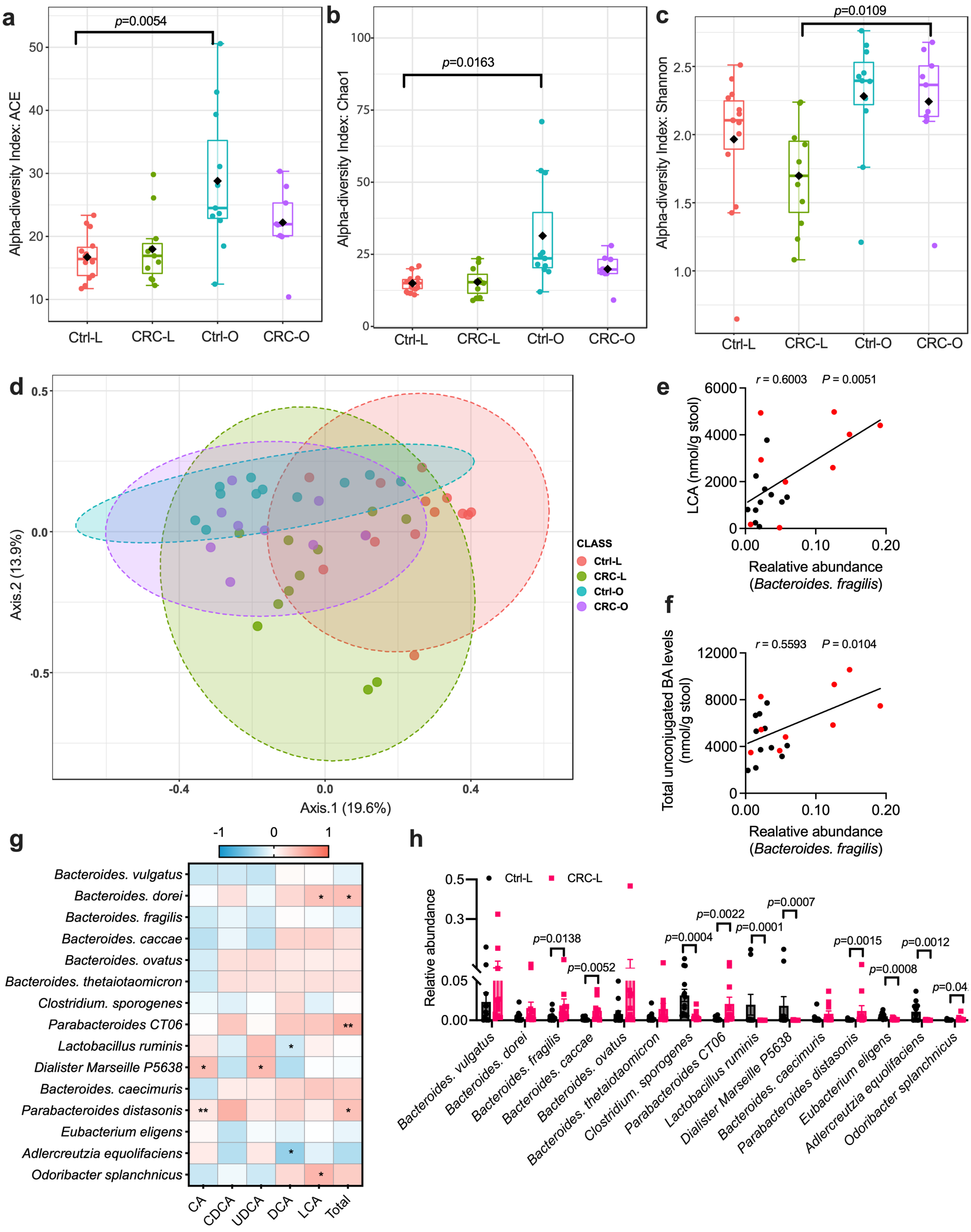
a
p=0.0054
Alpha-diversity Index: ACE
Ctrl-L
CRC-L
Ctrl-O
CRC-O
b
p=0.0163
Alpha-diversity Index: Chao1
c
p=0.0109
Alpha-diversity Index: Shannon
d
Axis.1 (19.6%)
Axis.2 (13.9%)
CLASS
Ctrl-L
CRC-L
Ctrl-O
CRC-O
e
r = 0.6003 P = 0.0051
LCA (nmol/g stool)
Realative abundance
(Bacteroides. fragilis)
f
r = 0.5593 P = 0.0104
Total unconjugated BA levels
(nmol/g stool)
Realative abundance
(Bacteroides. fragilis)
g
Bacteroides. vulgatus
Bacteroides. dorei
Bacteroides. fragilis
Bacteroides. caccae
Bacteroides. ovatus
Bacteroides. thetaiotaomicron
Clostridium. sporogenes
Parabacteroides CT06
Lactobacillus ruminis
Dialister Marseille P5638
Bacteroides. caecimuris
Parabacteroides distasonis
Eubacterium eligens
Adlercreutzia equolifaciens
Odoribacter splanchnicus
CA
CDCA
UDCA
DCA
LCA
Total
h
Relative abundance
Ctrl-L
CRC-L
p=0.0138
p=0.0052
p=0.0004
p=0.0022
p=0.0001
p=0.0007
p=0.0015
p=0.0008
p=0.0012
p=0.04

Supplementary Figure 1: BSH-producing *Bacteroides* were enriched in CRC patients with overweight, refers to Fig. 1

a-h Stool samples were collected from 45 individuals [14 for control lean group (Ctrl-L); 11 for CRC lean group (CRC-L); 11 for control overweight group (Ctrl-O); 9 for CRC overweight group (CRC-O)] for shotgun metagenomics and bile acid analysis. **a-c** α-diversity of the gut microbiota among four groups, as indicated by the ACE (**a**), Chao1 (**b**) and Shannon indices (**c**). One-way ANOVA with Tukey's post hoc test**. d** Partial least squares discriminant analysis (PLS-DA) using the Bray-Curtis distance. **e**, **f** Correlative analysis of *B. fragilis* with stool LCA (**e**) and total unconjugated bile acid levels (**f**) in subjects with overweight. Correlations between variables were assessed by linear regression analysis. Linear correction index R and *P* values were calculated with two-sided. **g** Heatmap of the correlation between variable gut microbiota and stool unconjugated bile acid levels in lean subjects. Correlation analysis were determined by Spearman's rank test with two-sided.. $^{*}P < 0.05$, $^{**}P < 0.01$. **h** Relative abundance of variable gut microbiota at species levels in lean subjects. Mann-Whitney *U* test with two-sided. Data are presented as mean values +/- SEM in a-c, h. Source data are provided as a Source Data file for Supplementary Figure 1.

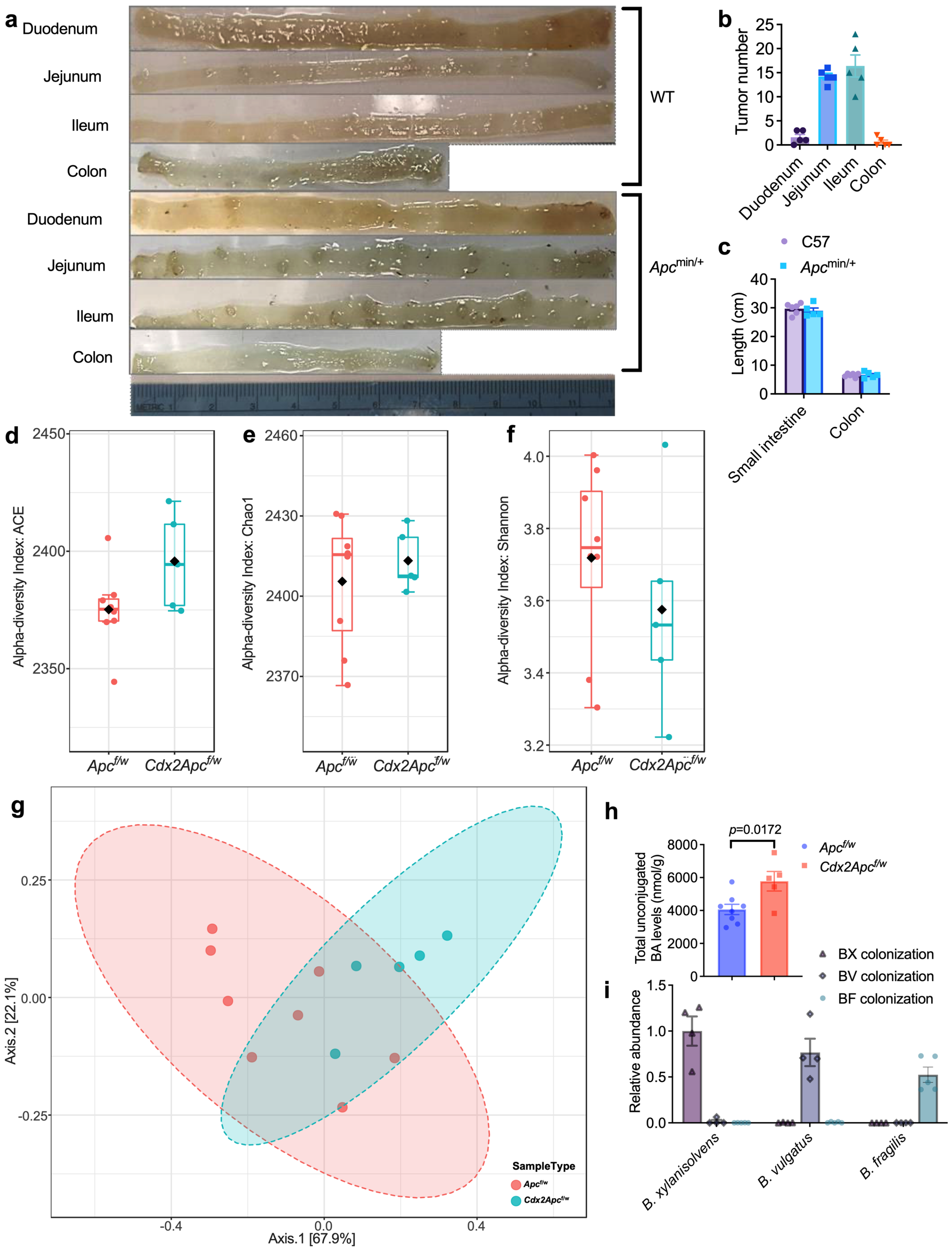
a
Duodenum
Jejunum
Ileum
Colon
WT
Duodenum
Jejunum
Ileum
Colon
Apc^min/+
b
Tumor number
Duodenum
Jejunum
Ileum
Colon
c
C57
Apc^min/+
Length (cm)
Small intestine
Colon
d
Alpha-diversity Index: ACE
e
Alpha-diversity Index: Chao1
f
Alpha-diversity Index: Shannon
Apc^f/w
Cdx2Apc^f/w
g
Axis.1 [67.9%]
Axis.2 [22.1%]
SampleType
Apc^f/w
Cdx2Apc^f/w
h
p=0.0172
Total unconjugated BA levels (nmol/g)
Apc^f/w
Cdx2Apc^f/w
i
BX colonization
BV colonization
BF colonization
Relative abundance
B. xylanisolvens
B. vulgatus
B. fragilis

Supplementary Figure 2: BSH-producing *Bacteroides* species are enriched in CRC mouse model, refers to Fig. 2

a-c C57BL/6J (WT) or *Apc*$^{min/+}$ mice were fed a HFD for 8 weeks (*n* = 6 mice for WT; *n* = 5 mice for *Apc*$^{min/+}$). **a**, Representative pictures of intestinal segments, indicating the tumor burden. **b** Tumor numbers in different segments of intestine from 5 *Apc*$^{min/+}$ mice. **c** Length of small intestine and colon. **d-h** *Apc*$^{f/w}$ and *Cdx2Apc*$^{f/w}$ mice were fed with HFD for 12 weeks, feces were collected for shotgun metagenomics (*n* = 6 mice/group). **d-f** α-diversity of the gut microbiota among four groups, as indicated by the ACE (**d**), Chao1 (**e**) and Shannon indices (**f**). **g** Partial least squares discriminant analysis (PLS-DA) using the Bray-Curtis distance. **h** Total unconjugated bile acid levels in the colon contents. Two-tailed Student's *t* test. **i** Relative abundance of *B. xylanisolvens*, *B. vulgatus* and *B. fragilis* after colonization with each species (*n* = 4 mice for *B. xylanisolvens* colonization; *n* = 4 mice for *B. vulgatus* colonization; *n* = 5 mice for *B. fragilis* colonization). Data are presented as mean values +/- SEM in b-f, h, i. Source data are provided as a Source Data file for Supplementary Figure 2.

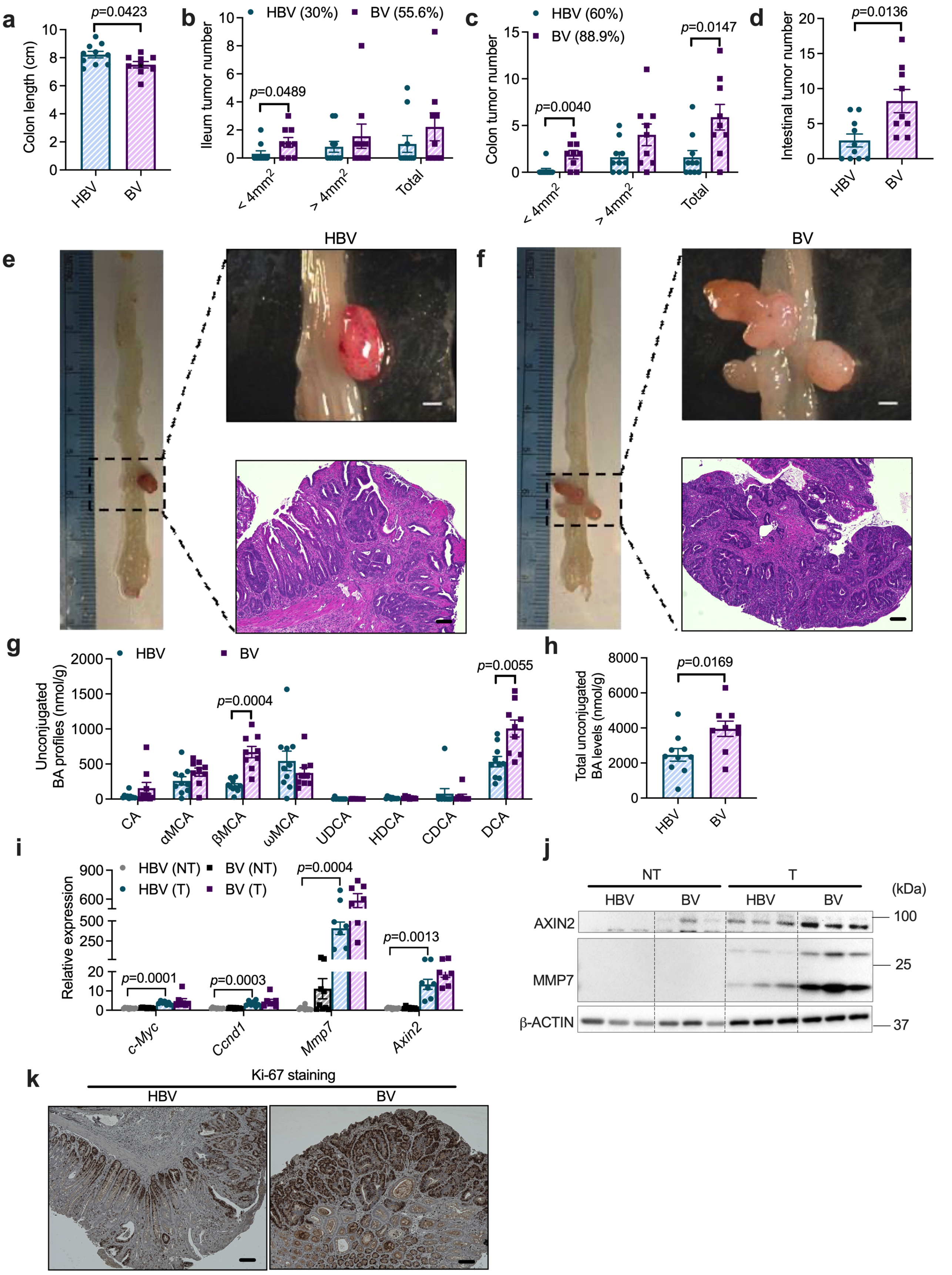
a
p=0.0423
Colon length (cm)
HBV
BV
b
HBV (30%)
BV (55.6%)
Ileum tumor number
p=0.0489
< 4mm2
> 4mm2
Total
c
HBV (60%)
BV (88.9%)
Colon tumor number
p=0.0040
p=0.0147
d
p=0.0136
Intestinal tumor number
e
HBV
f
BV
g
Unconjugated BA profiles (nmol/g)
p=0.0004
p=0.0055
CA
αMCA
βMCA
ωMCA
UDCA
HDCA
CDCA
DCA
h
p=0.0169
Total unconjugated BA levels (nmol/g)
i
HBV (NT)
BV (NT)
HBV (T)
BV (T)
Relative expression
p=0.0001
p=0.0003
p=0.0004
p=0.0013
c-Myc
Ccnd1
Mmp7
Axin2
j
NT
T
(kDa)
AXIN2
MMP7
β-ACTIN
100
25
37
k
Ki-67 staining

Supplementary Figure 3: *B. vulgatus* (BV) colonization aggreavtes CRC progression, refers to Fig. 3 **a-h** HFD-fed *Cdx2Apc*$^{f/w}$ mice were colonized with heat-killed HBV or BV for 12 weeks (*n* = 10 mice for HBV; *n* = 9 mice for BV). **a** Length of colon. **b**, **c** The ileum (**b**) and (**c**) colon tumor incidence, and tumor numbers with different sizes (<4 mm^2, >4 mm^2 and the sum of both). Mann-Whitney *U* test with two-sided. **d** Total tumor number in the intestine. Two-tailed Student's *t* test. **e**, **f** Representative pictures of colon (left), gross images of tumor (top right) in the colon and H&E staining (bottom right) of colon tumor sections. Scale bars: 1.5 mm (top right) and 100 μm (bottom right). **g** Unconjugated bile acid profiles in the colon contents. Mann-Whitney *U* test with two-sided. **h** Total unconjugated bile acid levels in the colon content. Two-tailed Student's *t* test. **i** Relative mRNA levels of WNT target genes in colon non-tumor (NT) and tumor (T) tissues (*n* = 10 for HBV-NT; *n* = 9 for BV-NT; *n* = 7 for HBV-T; *n* = 7 for BV-T;). Kruskal-Wallis test with Dunn's post hoc test. **j** WB data of proteins encoded by WNT target genes in colon non-tumor and tumor tissues (*n* = 3 independent samples/group). **k** Representative IHC staining of Ki-67, a proliferation marker (n = 7 independent slides for HBV; n = 9 independent slides for BV). Scale bars: 100 μm. Data are presented as mean values +/- SEM in a-d, g-i. Source data are provided as a Source Data file for Supplementary Figure 3.

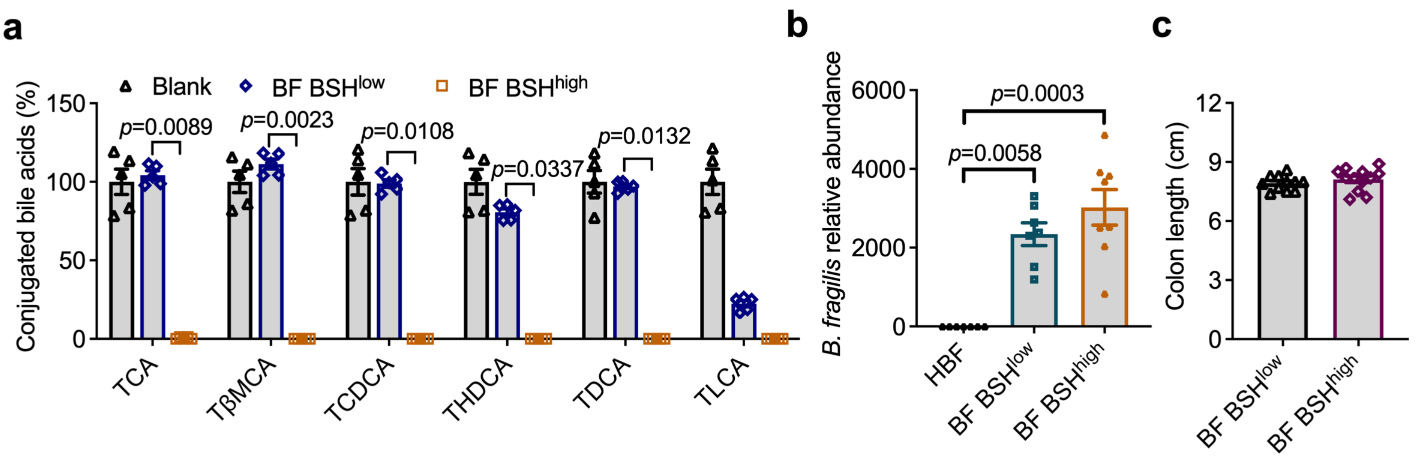

Supplementary Figure 4: Microbial BSH overexpression in *B. fragilis 638R* (BF638R), refers to Fig. 4 and 5

a Hydrolysis efficiency of conjugated bile acids in the culture medium of blank, WT *B. fragilis 638R* (BF BSH^{low}) or BSH-overexpressing *B. fragilis 638R* (BF BSH^{high}) (n = 5 technical replicates/group). Kruskal-Wallis test with Dunn's post hoc test. TCA: taurocholic acid; TβMCA: tauro-β-muricholic acid; TCDCA: taurochenodeoxycholic acid; THDCA: taurohyodeoxycholic acid; TDCA: taurodeoxycholic acid; TLCA: taurolithocholic acid. **b** HFD-fed *Cdx2Apc*$^{f/w}$ mice were transplanted with BF BSH^{low} or BF BSH^{high} for 2 weeks (n = 7 mice for HBF; n = 7 mice for BF BSH^{low}; n = 8 mice for BF BSH^{high}). Relative abundance of *B. fragilis* after colonization. One-way ANOVA with Tukey's post hoc test. **c** HFD-fed *Cdx2Apc*$^{f/w}$ mice were transplanted with BF BSH^{low} or BF BSH^{high} for 12 weeks (n = 11 mice for BF BSH^{low}; n = 12 mice for BF BSH^{high}). **c**, The length of colon. Data are presented as mean values +/- SEM in a-c. Source data are provided as a Source Data file for Supplementary Figure 4.

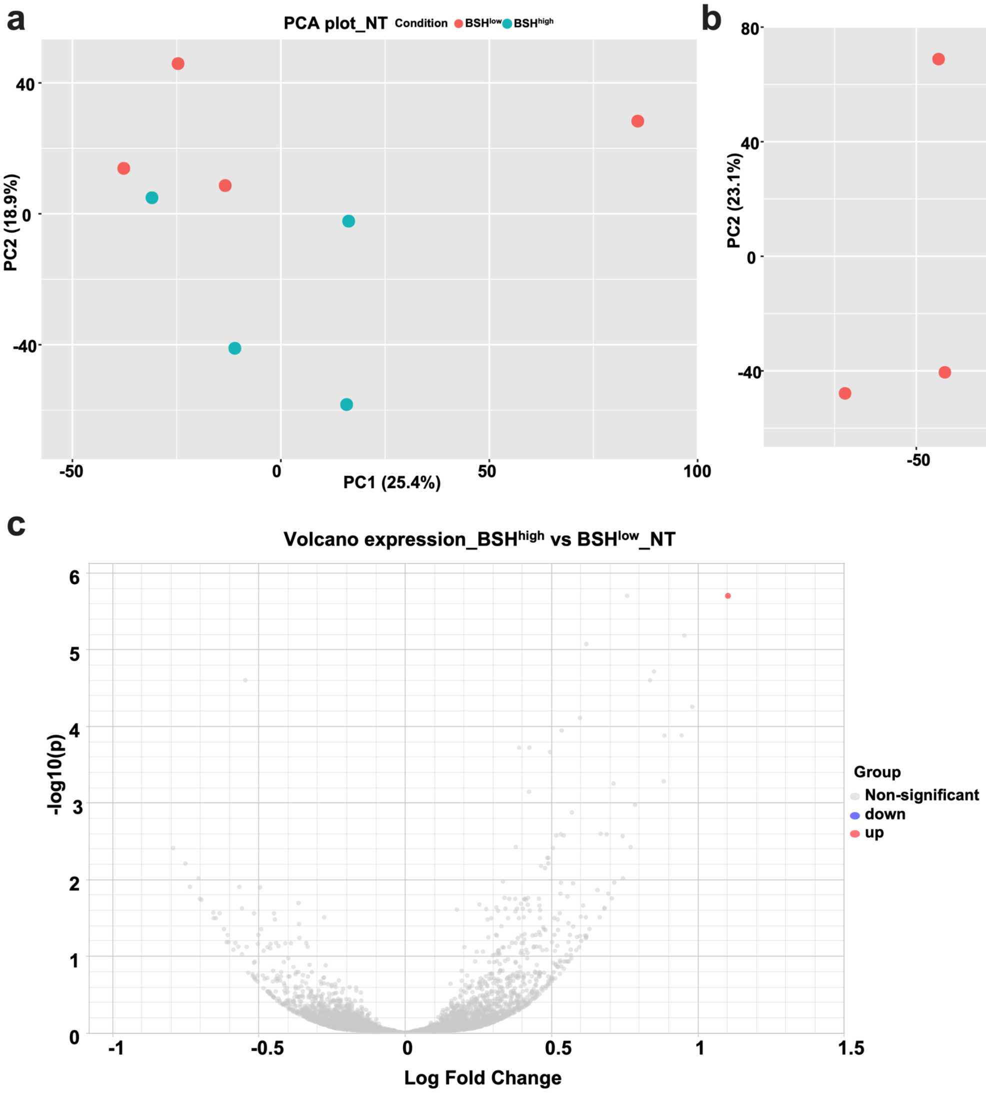

Supplementary Figure 5: Microbial BSH overexpression in *B. fragilis* change the gene expression profiles in colon cancer tissues, refers to Fig. 6

a-c HFD-fed *Cdx2Apc*$^{f/w}$ mice were transplanted with BF BSH^{low} or BF BSH^{high} for 12 weeks, from which non-tumor (T) and tumor (T) colon tissues were subjected for mRNA sequencing (pooled sample sizes: n = 4 independent samples for BF BSH^{low}_NT; n = 4 independent samples for BF BSH^{high}_NT; n = 4 independent samples for BF BSH^{low}_T; n = 3 independent samples for BF BSH^{high}_T). **a**, **b** Principal component analysis (PCA) for gene expression in NT (**a**) or T (**b**) tissues from BF BSH^{low} or BF BSH^{high} groups. **c** Volcano plot showing the expression profiles of genes in NT tissues of BF BSH^{low} and BF BSH^{high} groups. Red dot (upregulated) was identified as significantly changed gene with an adjust P value<0.05 and |logFC| > 1. Two-tailed Student's t test.

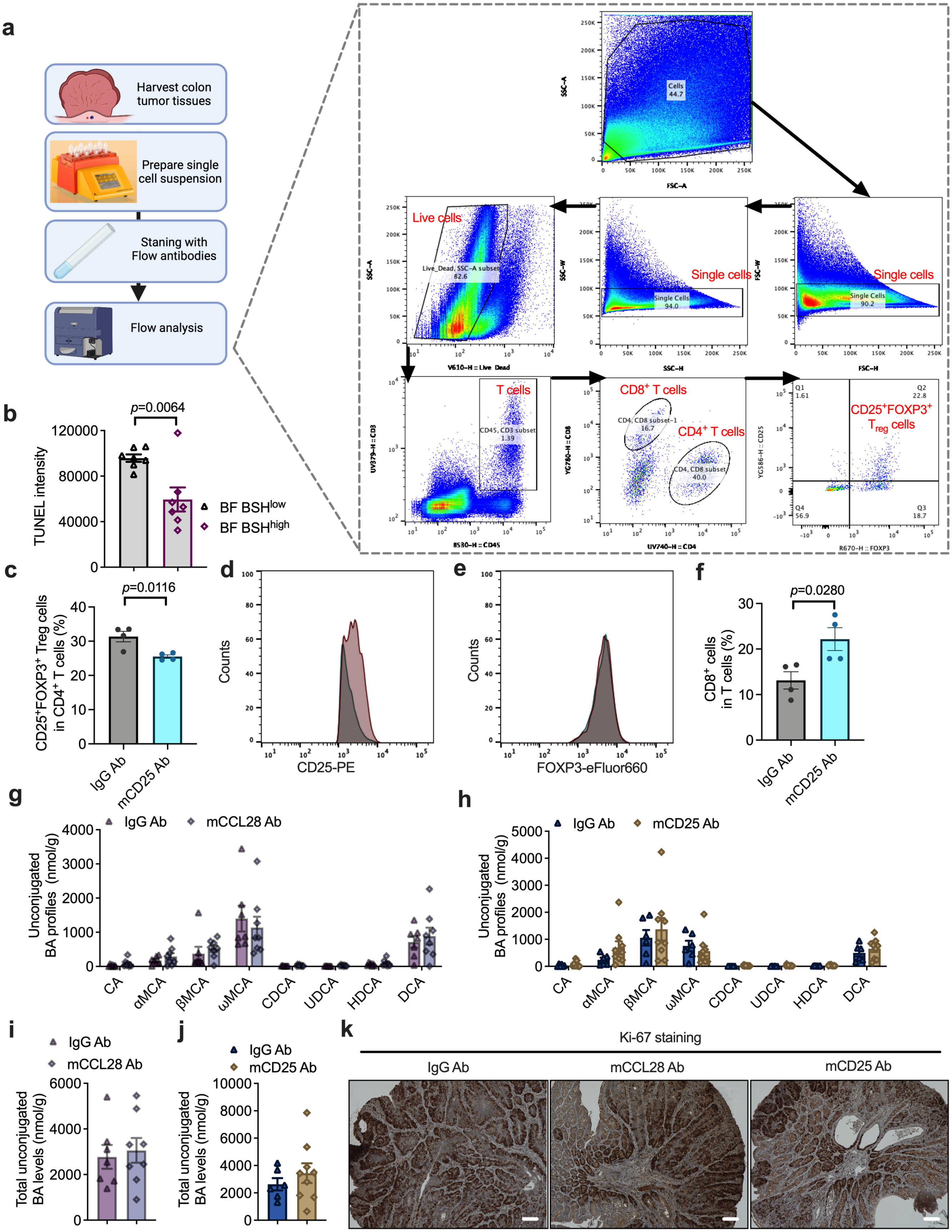
a
Harvest colon tumor tissues
Prepare single cell suspension
Staning with Flow antibodies
Flow analysis
Live cells
Single cells
Single cells
T cells
CD8+ T cells
CD4+ T cells
CD25+FOXP3+ Treg cells
b
p=0.0064
TUNEL intensity
BF BSHlow
BF BSHhigh
c
p=0.0116
CD25+FOXP3+ Treg cells in CD4+ T cells (%)
IgG Ab
mCD25 Ab
d
Counts
CD25-PE
e
Counts
FOXP3-eFluor660
f
p=0.0280
CD8+ cells in T cells (%)
IgG Ab
mCD25 Ab
g
IgG Ab
mCCL28 Ab
Unconjugated BA profiles (nmol/g)
CA
αMCA
βMCA
ωMCA
CDCA
UDCA
HDCA
DCA
h
IgG Ab
mCD25 Ab
Unconjugated BA profiles (nmol/g)
i
IgG Ab
mCCL28 Ab
Total unconjugated BA levels (nmol/g)
j
IgG Ab
mCD25 Ab
Total unconjugated BA levels (nmol/g)
k
Ki-67 staining
IgG Ab
mCCL28 Ab
mCD25 Ab

Supplementary Figure 6: CCL28-induced immunosuppressive effects contributed to *B. fragilis*-accelerated CRC progression, refers to Fig. 6 and 7

a Flow chart that establishes the flow cytometry procedures for analyzing immune properties in colon tumor tissues. Created with BioRender.com. **b** HFD-fed *Cdx2Apc*$^{f/w}$ mice were colonized with BF BSH^{low} or BF BSH^{high} for 12 weeks (n = 7 samples/group). TUNEL intensity. Two-tailed Student's t test. **c-f** *Cdx2Apc*$^{f/w}$ mice were fed with HFD for 10 weeks, and then the mice were colonized with BF BSH^{high} and injected with IgG or mCD25 Ab for another 2 weeks (n = 4 mice/group). The colon tumor tissues were harvested for flow cytometry analysis. **c** The portion of FOXP^{+}CD25^{+} T_{reg} cells in CD4^{+} T cells. Two-tailed Student's t test. **d**, **e** CD25 (**d**) and FOXP3 (**e**) intensity in FOXP^{+}CD25^{+} T_{reg} cells. **f** The portion of CD8^{+} T cells in total T cells. Two-tailed Student's t test. **g-k** HFD-fed *Cdx2Apc*$^{f/w}$ mice were fed with HFD for 6 weeks, and then colonized with BF BSH^{high} and injected (i.p.) with IgG, mCCL28 Ab or mCD25 Ab neutralizing antibody for another 6 weeks (n = 13 mice for IgG Ab; n = 8 mice for mCCL28 Ab; n = 9 mice for mCD25 Ab). **g**, **h** Unconjugated bile acid profiles in the colon contents of mCCL28 Ab (**g**) or mCD25 Ab (**h**)-treated mice. **i**, **j** Total unconjugated bile acid profiles in the colon contents of mCCL28 Ab (**i**) or mCD25 Ab (**j**)-treated mice. **k** Representative IHC staining of Ki-67, a proliferation marker (n = 10 independent slides for IgG Ab; n = 7 independent slides for mCCL28 Ab; n = 7 independent slides for mCD25 Ab). Scale bars: 100 μm. Data are presented as mean values +/- SEM in b, c, f-j. Source data are provided as a Source Data file for Supplementary Figure 6.

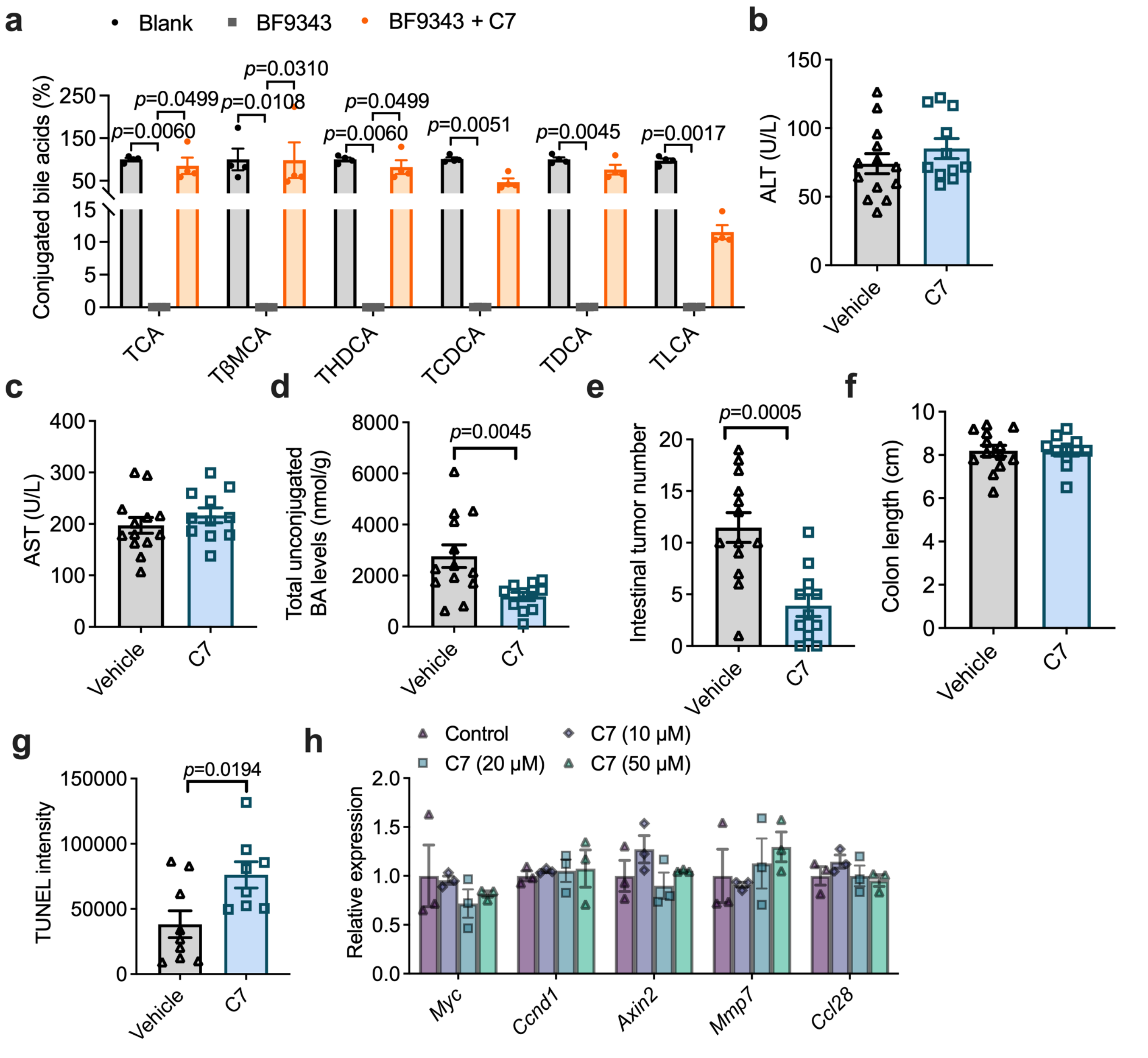

Supplementary Figure 7: Compound 7 (C7) postpones CRC progression by inhibiting microbial BSH activity, refers to Fig. 8

a *B. fragilis* 9343 (BF9343) were cultured with or without BSH inhibitor, C7 (10 μM), for overnight under conjugated bile acid administration (n = 4 technical replicates/group). Hydrolysis efficiency. Kruskal-Wallis test with Dunn's post hoc test. **b-g** Under BF BSH^{high} colonization, *Cdx2Apc*$^{f/w}$ mice were fed with HFD or HFD with C7 (10 mg/kg) for 12 weeks (n = 13 mice for Vehicle; n = 11 mice for C7). **b**, **c** Serum ALT (**b**) and AST (**c**) levels. **d** Total unconjugated bile acids in the colon contents. Two-tailed Student's t test. **e** Total tumor numbers in the intestine. Two-tailed Student's t test. **f** The length of colon. **g** TUNEL intensity. Two-tailed Student's t test. **h** *Cdx2Apc*$^{f/w}$ mice were fed with HFD for over 10 weeks, and then the colon tumors

were used for colon organoids isolation and further culture. Relative expression of β -catenin target genes and Ccl28 under different concentrations of C7 treatment for overnight (n = 3 technical replicates/group). Data are presented as mean values +/- SEM in a-h. Source data are provided as a Source Data file for Supplementary Figure 7.

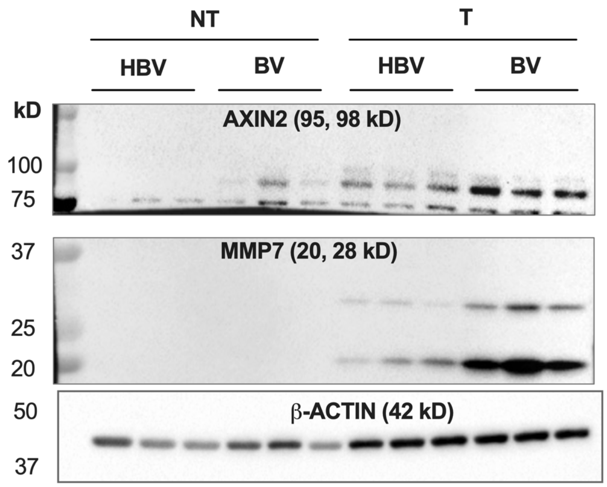

Supplementary Figure 8: Uncropped gels with markers, refers to Supplementary Fig. 3j

Supplementary Table 1: Demographic characteristics for human subjects

Patient ID	Group	BMI	TG (mmol/L)	TC (mmol/L)	ALT (U/L)	AST (U/L)	Tumor Primary Site
A1	Control-Lean	24.46	1.00	5.09	11.00	14.00	/
A2	Control-Lean	22.31	0.71	6.22	12.00	17.00	/
A3	Control-Lean	23.31	1.47	6.09	14.00	19.00	/
A4	Control-Lean	21.51	0.87	4.88	19.00	20.00	/
A5	Control-Lean	22.86	0.86	4.66	15.00	19.00	/
A6	Control-Lean	24.97	0.82	4.97	10.00	17.00	/
A7	Control-Lean	22.89	2.44	5.18	7.00	17.00	/
A8	Control-Lean	22.49	1.06	4.92	10.00	13.00	/
A9	Control-Lean	24.84	1.50	4.75	17.00	17.00	/
A10	Control-Lean	19.82	0.49	4.47	9.00	16.00	/
A11	Control-Lean	22.06	1.03	4.49	25.00	21.00	/
A12	Control-Lean	21.85	0.98	4.33	12.00	19.00	/
A13	Control-Lean	23.59	0.53	4.51	22.00	19.00	/
A14	Control-Lean	18.07	0.61	5.04	14.00	23.00	/
B1	Control-Overweight	25.01	1.21	4.53	16.00	17.00	/
B2	Control-Overweight	30.48	1.39	4.67	28.00	35.00	/
B3	Control-Overweight	25.69	1.18	5.23	20.00	25.00	/
B4	Control-Overweight	27.47	1.39	4.68	18.00	34.00	/
B5	Control-Overweight	27.64	1.27	4.67	16.00	17.00	/
B6	Control-Overweight	26.04	2.15	5.39	15.00	17.00	/
B7	Control-Overweight	31.24	2.87	5.52	17.00	21.00	/
B8	Control-Overweight	33.06	1.23	5.32	33.00	34.00	/
B9	Control-Overweight	32.76	1.21	5.28	22.00	29.00	/
B10	Control-Overweight	33.03	2.02	4.46	29.00	42.00	/
B11	Control-Overweight	29.71	4.16	7.64	40.00	63.00	/
C1	CRC-Lean	22.65	3.84	5.53	27.00	22.00	Left Colon
C2	CRC-Lean	24.77	1.48	4.73	15.00	19.00	Left Colon
C3	CRC-Lean	19.56	0.76	3.37	6.00	12.00	Right Colon
C4	CRC-Lean	21.88	1.35	3.85	8.00	10.00	Rectum
C5	CRC-Lean	23.46	3.56	6.23	19.00	19.00	Left Colon
C6	CRC-Lean	22.86	1.62	3.24	20.00	19.00	Rectum
C7	CRC-Lean	24.09	0.99	4.41	9.70	11.80	Right Colon
C8	CRC-Lean	19.90	0.59	4.70	16.00	18.00	Rectum
C9	CRC-Lean	20.96	0.78	2.95	10.00	14.00	Left Colon
C10	CRC-Lean	22.99	1.17	5.10	11.00	15.00	Left Colon
C11	CRC-Lean	19.78	0.58	3.71	19.00	28.00	Right Colon

D1	CRC-Overweight	26.04	1.09	7.13	15.00	17.00	Left Colon
D2	CRC-Overweight	27.06	1.80	3.71	9.00	14.00	Right Colon
D3	CRC-Overweight	28.51	0.85	5.55	17.00	24.00	Right Colon
D4	CRC-Overweight	26.73	2.38	4.31	18.00	33.00	Left Colon
D5	CRC-Overweight	25.91	1.44	4.07	14.00	25.00	Left Colon
D6	CRC-Overweight	32.51	1.68	6.61	18.00	18.00	Left Colon
D7	CRC-Overweight	25.95	1.41	4.93	13.00	13.00	Left Colon
D8	CRC-Overweight	30.07	1.40	4.31	13.00	16.00	Rectum
D9	CRC-Overweight	32.84	1.09	5.72	10.00	15.00	Left Colon

Supplementary Table 2: Sequences of the real time PCR primers

Primers	Forward	Reverse
**Mouse**		
Ccnd1	GCGTACCCTGACACCAATCTC	CTCCTCTTCGCACTTCTGCTC
c-Myc	ATGCCCCTCAACGTGAACTTC	CGCAACATAGGATGGAGAGCA
Mmp7	CTTACCTCGGATCGTAGTGGA	CCCCAACTAACCCTCTTGAAGT
Axin2	ATGAGTAGCGCCGTGTTAGTG	GGGCATAGGTTTGGTGGACT
Ccl28	GTGTGTGGCTTTTCAAACCTCA	TGCATGAACTCACTCTTTCCAG
Actin	GGCTGTATTCCCCTCCATCG	CCAGTTGGTAACAATGCCATGT
**Bacteria**		
Universal	TGGAGAGTTTGATCCTGGCTCAG	TACCGCGGCTGCTGGCAC
B. fragilis	CGGATGCCATTGATAAAGTAGG	CTGGAAGCAAGCACATTAGC
B. vulgatus	CGGGCTTAAATTGCAGATGA	CATGCAGCACCTTCACAGAT
BF9343_1433 (Bsh)	GAGTGGTCTATTCCTTGGGG	GGATCGCTGCAGGTGCAC
B. xylanisolvens	GTGAAGGTGCTGCATGGTTG	TGGGATTAGCATCCTGTCGC

Supplementary Table 3: Bacterial strains and plasmids used in this study

Strains	Genotype
B. vulgatus	appendix abscess
B. fragilis 638R	clinical isolate, Rif^r
B. fragilis ATCC 25285 (NCTC9343)	appendix abscess
BER-154	638R carrying pNBU2-bla-*ermGb*, Rif^r Erm^r
BER-182	638R carrying pER-300 Rif^r Erm^r
E. coli S17-1 λpir	Strain with the RK2 *tra* genes for conjugative transfer integrated in the chromosome (*RP4-2-Tc::Mu-Km::Tn7, pro, res−mod+*, Tp^r Sm^r) λ*pir* lysogen)
**Plasmids**	
pNBU2-*bla-ermGb*	NBU2 integrase (*intN2*) based genomic insertion vector derived from pKNOCK-*bla-ermGb* inserts into NBU2 *att1* or *att2* sites of $tRNA^{ser}$, (Amp^r) Erm^r
pER-300	A 1,569 bp DNA fragment containing entire *bsh* operon (BF9343_1433) was cloned into the XbaI/PstI sites of pNBU2-*bla-ermGb*, (Amp^r) Erm^r

Erm^r: erythromycin resistance; Rif^r: rifampicin resistance; Amp^r: Ampicillin resistance. Parenthesis indicates antibiotic resistance expression in *E. coli*. ATCC: American Type Culture Collection. NCTC: National Collection of Type Cultures, England.
