# Supplementary material for: Bile salt hydrolase in non-enterotoxigenic Bacteroides potentiates colorectal cancer
Source: Nat Commun. 2023 Feb 10;14:755. doi: 10.1038/s41467-023-36089-9 (PMC9918522; doi:10.1038/s41467-023-36089-9)
Supplement: Supplementary file 2 — Description of Additional Supplementary Files [file 41467_2023_36089_MOESM2_ESM.pdf]

Description of Additional Supplementary Files

Supplementary Data 1: human shotgun metagenomics outputs

Supplementary Data 2: Mouse shotgun metagenomics outputs

Supplementary Data 3: Bulk mRNA-seq output
