# Supplementary material for: Bile salt hydrolase in non-enterotoxigenic Bacteroides potentiates colorectal cancer
Source: Nat Commun. 2023 Feb 10;14:755. doi: 10.1038/s41467-023-36089-9 (PMC9918522; doi:10.1038/s41467-023-36089-9)
Supplement: Supplementary file 6 — Reporting Summary [file 41467_2023_36089_MOESM6_ESM.pdf]

nature portfolio

Corresponding author(s): Frank J. Gonzalez

Last updated by author(s): 1/12/2022

Reporting Summary

Nature Portfolio wishes to improve the reproducibility of the work that we publish. This form provides structure for consistency and transparency in reporting. For further information on Nature Portfolio policies, see our Editorial Policies and the Editorial Policy Checklist.

Statistics

For all statistical analyses, confirm that the following items are present in the figure legend, table legend, main text, or Methods section.

n/a	Confirmed	
☐	☒	The exact sample size (*n*) for each experimental group/condition, given as a discrete number and unit of measurement
☐	☒	A statement on whether measurements were taken from distinct samples or whether the same sample was measured repeatedly
☐	☒	The statistical test(s) used AND whether they are one- or two-sided<br>*Only common tests should be described solely by name; describe more complex techniques in the Methods section.*
☐	☒	A description of all covariates tested
☐	☒	A description of any assumptions or corrections, such as tests of normality and adjustment for multiple comparisons
☐	☒	A full description of the statistical parameters including central tendency (e.g. means) or other basic estimates (e.g. regression coefficient) AND variation (e.g. standard deviation) or associated estimates of uncertainty (e.g. confidence intervals)
☐	☒	For null hypothesis testing, the test statistic (e.g. *F*, *t*, *r*) with confidence intervals, effect sizes, degrees of freedom and *P* value noted<br>*Give P values as exact values whenever suitable.*
☒	☐	For Bayesian analysis, information on the choice of priors and Markov chain Monte Carlo settings
☒	☐	For hierarchical and complex designs, identification of the appropriate level for tests and full reporting of outcomes
☐	☒	Estimates of effect sizes (e.g. Cohen's *d*, Pearson's *r*), indicating how they were calculated

Our web collection on statistics for biologists contains articles on many of the points above.

Software and code

Policy information about availability of computer code

Data collection	1. For human shotgun metagenomics, HiSeq 250 PE clustering suite was adopted to cluster the index coded samples by the cBot clustering generation system, while the Illumina Nova 6000 platform was chosen to sequence, and 150-bp paired-end reads were generated. For mouse shotgun metagenomics, the samples were submitted to Pennsylvania State University Genomics Core Facility (University Park, PA) for NextSeq Mid-Output 150 x 150 paired end sequencing.<br>2. Bile acid metabolites were identified and quantified by an Acquity® UPLC/G2Si QTOFMS system (Waters Corp.) with an electrospray ionization (ESI) source.<br>3. Tumor numbers were counted under a stereo microscope equipped with a Jenoptik camera and television monitor.<br>4. All IHC images were taken by Keyence BZ-X710 under the same settings.<br>5. mRNA-Seq samples were pooled and sequenced on NextSeq2000 using Illumina TruSeq Stranded mRNA Library Prep and paired-end sequencing by the National Cancer Institute Core Sequencing Facility. Reads of the samples were trimmed for adapters and low-quality bases using Cutadapt before alignment with the reference genome (mm10) and the annotated transcripts using STAR. The mapping statistics are calculated using Picard. Library complexity is measured in terms of unique fragments in the mapped reads using Picard's MarkDuplicate utility.<br>6. Flow cytometry analysis were conducted by LSRFortessa SORP I (BD Biosciences).<br>7. Other Data were collected with Microsoft Excel version 16.68.
Data analysis	1. Clean metagenomic sequence reads were analyzed using the Kraken 2 taxonomic sequence classification approach on standard Kraken 2 version 2.0.8-beta60 comprising of all complete bacterial, viral and archeal genomes in RefSeq. Abundance of the various species was estimated using Bracken. For functional gene analysis, a local Diamond database was created using all the Kegg genes involved in bile acid metabolism. Filtered reads were aligned to the local database with Diamond version 0.9.36 with a search flag, sensitive.<br>2. Bile acid quantification was analyzed by TargetLynx in MassLynx Version 4.2.

3. The tumor sizes were further analyzed by a ProgRes CapturePro version 2.10.0.1.
4. Signal intensity of IHC staining was measured on Fiji software (ImageJ version 1.53t).
5. For mRNA sequencing, gene expression quantification analysis was performed for all samples using STAR/RSEM tools. The following analysis was performed in NetworkAnalyst platform (https://www.networkanalyst.ca).
6. Flow cytometry data analysis was performed with Flow Jo Version 10 (BD Biosciences).
7. GraphPad Prism version 9.0 was used to analyze the statistics.

For manuscripts utilizing custom algorithms or software that are central to the research but not yet described in published literature, software must be made available to editors and reviewers. We strongly encourage code deposition in a community repository (e.g. GitHub). See the Nature Portfolio guidelines for submitting code & software for further information.

Data

Policy information about availability of data

All manuscripts must include a data availability statement. This statement should provide the following information, where applicable:

- Accession codes, unique identifiers, or web links for publicly available datasets
- A description of any restrictions on data availability
- For clinical datasets or third party data, please ensure that the statement adheres to our policy

Bulk mRNA-seq output was listed in Supplementary Data 3 and the original sequencing data set has been deposited in GEO database under accession code GSE190298 (https://www.ncbi.nlm.nih.gov/geo/query/acc.cgi?acc=GSE190298). Mouse and human shotgun metagenomics outputs were listed in Supplementary Data 1 and 2, and the sequencing datasets have been uploaded to the public database in National Library of Medicine under accession codes PRJNA786913 for mouse and PRJNA881471 for human. https://www.ncbi.nlm.nih.gov/bioproject/PRJNA786913; https://www.ncbi.nlm.nih.gov/bioproject/PRJNA881471. Source data are provided with this paper.

Human research participants

Policy information about studies involving human research participants and Sex and Gender in Research.

Reporting on sex and gender	We collected stool samples from both male and female subjects. The information regarding the sex of each participant was collected by self-reporting via a questionnaire.
Population characteristics	Participants were aged 28-86 years, Chinese, with a BMI of 18.07-33.06 kg/m2. Participants in CRC group were pathologically confirmed diagnosis of colorectal cancer without other combined tumors and distant metastasis. Participants in Control group had no no history of inflammatory bowel disease (Crohn's disease, ulcerative colitis), other malignant tumors or bowel resection. More detailed information related to health status (i.e., age, sex, BMI and biochemical indicators) were collected (Supplementary Table 1).
Recruitment	Healthy individuals and individuals who were diagnosed with CRC by colonoscopy and/or undergone colorectal surgery at the Peking University Third Hospital were enrolled. According to the cut-offs of BMI provided by the World Health Organization (WHO), individuals of BMI≥25 kg/m2 belong to overweight group, and individuals of BMI<25 kg/m2 belong to lean group. Exclusion criteria are as follows: (1) Patients used antibiotics, probiotics preoperative four weeks; (2) Patients with a history of inflammatory bowel disease (Crohn's disease, ulcerative colitis); (3) Patients with a history of other malignant tumors; (4) Patients with a history of intestinal resection and (5) Patients receiving neoadjuvant therapy before surgery. Finally, 45 individuals (14 for control lean group; 11 for control overweight group; 11 for CRC lean group; 9 for CRC overweight group) were included in the study. All patients have signed informed consent forms prior to enrollment in the study. Participants can get their intestinal macrobiota composition data and fare compensation.
Ethics oversight	This study conformed to the ethical principles outlined by the Declaration of Helsinki and ethical approval for this study was obtained from the Ethics Committee of Peking University Third Hospital (IRB00006761-LM2022557).

Note that full information on the approval of the study protocol must also be provided in the manuscript.

Field-specific reporting

Please select the one below that is the best fit for your research. If you are not sure, read the appropriate sections before making your selection.

- [x] Life sciences
- [ ] Behavioural & social sciences
- [ ] Ecological, evolutionary & environmental sciences

For a reference copy of the document with all sections, see nature.com/documents/nr-reporting-summary-flat.pdf

Life sciences study design

All studies must disclose on these points even when the disclosure is negative.

Sample size	There were at least 3 samples or replicates for each experiment in this manuscript. Especially for animal experiments, there were over 5 mice per group in order to obtain statistical significance. Sample size was estimated on the basis of sample availability and previous studies.

Data exclusions	No data was exclued.
Replication	All data representative of three or more independent experiments, as stated in the figure legends.
Randomization	Human subjects were randomly selected. Healthy individuals and individuals who were diagnosed with CRC by colonoscopy and/or undergone colorectal surgery at the Peking University Third Hospital were enrolled as Control and CRC groups. According to the cut-offs of BMI provided by the World Health Organization (WHO), individuals of BMI≥25 kg/m2 belong to overweight group, and individuals of BMI<25 kg/m2 belong to lean group.<br>6-8 week old mice were divided at random into experimental groups, with at least 5 mice per group, and the groups did not present differences in age or body weights before the treatments.
Blinding	Investigators were blinded to group allocation during data collection and analysis. But during the treatment of live animals it was not blinded, as the treatment of each mouse would need to be known to the person handling the mice.

Reporting for specific materials, systems and methods

We require information from authors about some types of materials, experimental systems and methods used in many studies. Here, indicate whether each material, system or method listed is relevant to your study. If you are not sure if a list item applies to your research, read the appropriate section before selecting a response.

Materials & experimental systems

n/a	Involved in the study
☐	☒ Antibodies
☒	☐ Eukaryotic cell lines
☒	☐ Palaeontology and archaeology
☐	☒ Animals and other organisms
☒	☐ Clinical data
☒	☐ Dual use research of concern

Methods

n/a	Involved in the study
☒	☐ ChIP-seq
☐	☒ Flow cytometry
☒	☐ MRI-based neuroimaging

Antibodies

Antibodies used	For Western blot:<br>Rabbit anti-mouse polyclonal anti-AXIN2, Abcam, Cat#ab32197, Lot#GR3363772-2, 1:1000 dilution<br>Rabbit anti-mouse monoclonal anti-ACTB [13E5], Cell signaling, Cat#4670, Lot#15, 1:1000 dilution<br>Rabbit anti-mouse monoclonal anti-MMP7 [D4H5], Cell signaling, Cat#3801, Lot#2, 1:1000 dilution<br>Rabbit anti-mouse monoclonal anti-CCL28 [G-2], Santa Cruz, Cat# sc-376654, Lot#H0416, 1:500 dilution<br>For Flow cytometry:<br>Rat anti-mouse monoclonal anti-CD45 [30-F11, FITC], eBioscience, Cat#11-0451-82, Lot#2015744, 1:100 dilution<br>Hamster anti-mouse monoclonal anti-CD3e [145-2C11, BUV395], BD Biosciences, Cat#563565, Lot#9204644, 1:100 dilution<br>Rat anti-mouse monoclonal anti-CD4 (RM4-5, BUV737), BD Biosciences, Cat#612843, Lot#8225756, 1:100 dilution<br>Rat anti-mouse monoclonal anti-CD8a [53-6.7, PE-Cy7], BD Biosciences, Cat#552877, Lot#0300017, 1:100 dilution<br>Rat anti-mouse monoclonal anti-CD25 [3C7, PE], BD Biosciences, Cat#553075, Lot#1102788, 1:100 dilution<br>Rat anti-mouse monoclonal anti-FOXP3 [PCH101], eFluor 660, eBioscience, Cat#50-4776-42, Lot#2272756, 1:50 dilution<br>For in-vivo neutralization:<br>InVivoMAb rat IgG1 isotype control, anti-horseradish peroxidase [HRPN], Bio X Cell, Cat#BP0088, Lot#724921M1, 25 or 50 mg/kg, twice a week<br>Rat anti-mouse monoclonal anti-CCL28 [134306], R&D systems, Cat#MAB533, 50mg/kg, twice a week<br>InVivoPlus anti-mouse CD25 (IL-2Rα) [PC61.5.3], Bio X Cell, Cat#BP0012, Lot#795321D1, 25 mg/kg, twice a week<br>For IHC staining:<br>Recombinant Anti-Ki67 antibody [SP6], Abcam, Cat#ab16667, 1:100 dilution
Validation	Rabbit anti-mouse polyclonal anti-AXIN2, Citation: 10.1038/s41419-021-03733-5.<br>Rabbit anti-mouse monoclonal anti-ACTB, Citation: 10.1038/s41586-018-0729-3.<br>Rabbit anti-mouse monoclonal anti-MMP7 [D4H5], Citation: 10.1016/j.chom.2019.03.012.<br>Rabbit anti-mouse monoclonal anti-CCL28 [G-2], Citation: 10.18632/aging.102239.<br>Rat anti-mouse monoclonal anti-CD45 [30-F11, FITC], Citation: 10.1038/s41467-020-15129-8.<br>Hamster anti-mouse monoclonal anti-CD3e [145-2C11, BUV395], Citation: 10.1016/s1074-7613(00)80275-7.<br>Rat anti-mouse monoclonal anti-CD4 (RM4-5, BUV737), Citation: 10.1084/jem.190.10.1517.<br>Rat anti-mouse monoclonal anti-CD8a [53-6.7, PE-Cy7], Citation: 10.1126/science.1063564.<br>Rat anti-mouse monoclonal anti-CD25 [3C7, PE], Citation: 10.1016/0092-8674(93)90152-g.<br>Rat anti-mouse monoclonal anti-FOXP3 [PCH101, eFluor 660], Citation: 10.1038/s41591-020-0761-3.<br>InVivoMAb rat IgG1 isotype control, anti-horseradish peroxidase [HRPN], Citation: 10.1038/nature16962.<br>Rat anti-mouse monoclonal anti-CCL28 [134306], Citation: 10.4049/jimmunol.1100402.<br>InVivoPlus anti-mouse CD25 (IL-2Rα) [PC61.5.3], Citation: 10.1038/nature16962.

Recombinant Anti-Ki67 antibody [SP6], Citation: 10.1002/hep.31987.

Animals and other research organisms

Policy information about studies involving animals; ARRIVE guidelines recommended for reporting animal research, and Sex and Gender in Research

Laboratory animals	For this study, 6- to 8-week-old C57BL/6J (WT), C57BL/6J-ApcMin/J (Apcmin/+, The Jackson Laboratory, #002020), C57BL/6-Apctm1Tyj/J (Apcf/w, The Jackson Laboratory, #009045) and B6.Cg-Tg (CDX2-cre)101Erf (The Jackson Laboratory, #009350) were applied. To obtain colon-specific APC mutation, Apcf/w the mice were crossed with B6.Cg-Tg (CDX2-cre)101Erf mice harboring the Cre recombinase under control of the Cdx2 promoter. The mice were randomly divided into different groups, housed 3–5 per cage and maintained under standard laboratory conditions (the light from 08:00 to 20:00, the temperature at 21–24°C and the humidity at 40–70%) with free access to a 60% HFD (Bio-Serv, Cat#S3282) and water at the NCI, NIH.<br>For screening the gut microbiota during CRC progression, 6- to 8-week-old male Apcf/w and Cdx2Apcf/w mice were fed a 60% HFD for 12 weeks. For bacteria colonization, 6- to 8-week-old male Cdx2Apcf/w mice were given three-day antibiotic cocktail water [1 mg/mL neomycin (Millipore Sigma, #N1876), 1 mg/mL streptomycin (Millipore Sigma, #S19137), and 1 mg/mL bacitracin (Millipore Sigma, #B0125)], and then the mice were treated with 2×108 CFUs of bacteria in 200 µL of sterile anaerobic PBS by gavage every three days. For short-term CCL28 blockage, 6- to 8-week-old male Cdx2Apcf/w mice were pretreated with 60% HFD (Bio-Serv, #S3282) for 10 weeks, and then were colonized with BF BSHhigh and received injections of 50 mg/kg IgG (Bio X Cell, #BP0088) or mCCL28 Ab (R&D systems, #MAB533) twice a week for another 2 weeks. For long-term CCL28/Treg cell blockage, 6- to 8-week-old male Cdx2Apcf/w mice were pretreated with 60% HFD for 6 weeks, and then were colonized with BF BSHhigh and received injections of IgG (25 mg/kg), mCCL28 Ab (50 mg/kg) or mCD25 Ab (25 mg/kg, Bio X Cell, #BP0012) twice a week for another 6 weeks. For short-term C7 treatment, 6- to 8-week-old male Cdx2Apcf/w mice were pretreated with 60% HFD for 10 weeks, and then were colonized with BF BSHhigh and fed a 60% HFD or 60% HFD containing 1 mg/g C7 (TargetMol), which equates to 10 mg/kg/d consumption. For long-term C7 treatment, 6- to 8-week-old male Cdx2Apcf/w mice were colonized with BF BSHhigh and fed a 60% HFD or 60% HFD containing C7 for 12 weeks. The detailed mouse number for each experiment was listed in the relevant figure legend.
Wild animals	This study did not involve wild animals.
Reporting on sex	In epidemiological studies, male sex has consistently shown strong associations with CRC incidence (PMID: 31631858). Male mice were applied in this study.
Field-collected samples	Field-collected samples were not used in this study.
Ethics oversight	All mice were maintained in a specific pathogen-free (SPF) environment, and the animal protocols (protocol numbers: LM-027 and LM-092) for mouse experimentation were approved by the National Cancer Institute Animal Care and Use Committees of National Institutes of Health (NIH).

Note that full information on the approval of the study protocol must also be provided in the manuscript.

Flow Cytometry

Plots

Confirm that:

☒ The axis labels state the marker and fluorochrome used (e.g. CD4-FITC).

☒ The axis scales are clearly visible. Include numbers along axes only for bottom left plot of group (a 'group' is an analysis of identical markers).

☒ All plots are contour plots with outliers or pseudocolor plots.

☒ A numerical value for number of cells or percentage (with statistics) is provided.

Methodology

Sample preparation	Colons tumors were harvested and flushed with cold PBS. The collected colon tumors were further washed in cold colon buffer and cut into small fragments that were transferred into gentleMACS™ C Tubes with 10 mL pre-warm colon buffer containing 100 U/mL collagenase E. Cell dissociation was performed with gentleMACS™ Octo Dissociator. Isolated single cells (~1×106) were resuspended in FACS buffer and stained with LIVE/DEADTM fixable yellow dye and cell surface markers, including CD45, CD3e, CD4, CD8a, CD25. Before further staining with nuclear FOXP3, the cells were fixed and permeabilized with eBioscienceTM FOPX3/transcription factor staining buffer set.
Instrument	Flow cytometry analysis were conducted by LSRFortessa SORP I (BD Biosciences).
Software	Flow cytometry analysis were conducted by LSRFortessa SORP I. Flow cytometry data analysis was performed with Flow Jo Version 10.
Cell population abundance	No sorting was applied in this study.

Gating strategy

To designate single cells, FSC/SSC gating was utilized first, and then Live/Dead V610 was used to gate live single cells. CD45+CD3+ cells were characterized as T cell populations in live single cells. CD4+CD8- T cells were classified as CD4+ T cells. CD25 and FOXP3 were used as gating markers in CD4+ T cells to identify CD25+FOXP3+ Treg cells. To define "negative" or "positive" for each marker, single stained samples of non-stained, live/dead, CD3e, CD45, CD8, CD4, CD25, and FOXP3 were employed.

☒ Tick this box to confirm that a figure exemplifying the gating strategy is provided in the Supplementary Information.
